# A Pilot Investigation of Circulating miRNA Expression in Individuals Exposed to Aluminum and Welding Fumes

**DOI:** 10.3390/cimb47050306

**Published:** 2025-04-26

**Authors:** Gözde Öztan, Halim İşsever, Tuğçe İşsever, Fatma Oğuz, Sevgi Canbaz, Canan Küçükgergin, Kazım Yalçın Arga

**Affiliations:** 1 Department of Medical Biology, Istanbul Faculty of Medicine, Istanbul University, Topkapı, 34093 Istanbul, Turkey; oguzsf@istanbul.edu.tr; 2 Department of Public Health, Istanbul Faculty of Medicine, Istanbul University, Topkapı, 34093 Istanbul, Turkey; hissever@istanbul.edu.tr (H.İ.); sevgi.canbaz@istanbul.edu.tr (S.C.); 3 Turkish Health Institutes Presidency (TUSEB), 34718 Istanbul, Turkey; tugceissever@gmail.com; 4 Department of Biochemistry, Istanbul Faculty of Medicine, Istanbul University, Topkapı, 34093 Istanbul, Turkey; ckgergin@istanbul.edu.tr; 5 Department of Bioengineering, Faculty of Engineering, Marmara University, 34722 Istanbul, Turkey; kazim.arga@marmara.edu.tr

**Keywords:** expression, miRNA, qRT-PCR, aluminum, welding fume, heavy metal exposure

## Abstract

The objectives of this study comprise the identification of key miRNAs and their target genes associated with severe tolerance in individuals exposed to aluminum and welding fumes, and the elucidation of the underlying regulatory mechanisms. In this study, the levels of seven miRNAs (hsa-miR-19a-3p, hsa-miR-130b-3p, hsa-miR-25-3p, hsa-miR-363-3p, hsa-miR-92a-3p, hsa-miR-24-3p, and hsa-miR-19b-3p) were analyzed using both hsa-miR-16-5p and RNU6 (U6 snRNA) as reference miRNAs to validate normalization reliability. The qRT-PCR method was used on blood serum samples from 16 workers who were exposed to aluminum, 16 workers who were exposed to welding fumes, and 16 healthy controls who were not exposed to aluminum or welding fumes. We determined heavy metal levels from serum samples of workers exposed to aluminum and welding fumes and control groups using the ICP-OES method. The expression levels of hsa-miR-19a-3p and hsa-miR-19b-3p in aluminum-exposed and control groups were found to be statistically significant (*p* < 0.05). When workers exposed to welding fumes were compared with the those in the control groups, the expression levels of hsa-miR-19a-3p, hsa-miR-130b-3p, hsa-miR-92a-3p, and hsa-miR-24-3p were observed to be statistically significant (*p* < 0.05). This study shows that the identification of miRNAs and target genes in different biological functions and pathways plays an important role in understanding the molecular mechanisms of responses to heavy metal toxicity. We share the view that the study will make a significant contribution to the literature in that seven candidate miRNAs can be used as possible biomarkers for exposure to aluminum and welding fumes in humans.

## 1. Introduction

### 1.1. Heavy Metal Exposure and Health Risks

Heavy metal contamination is a growing environmental and public health concern due to its adverse effects on human health. Among toxic metals, chromium (Cr), copper (Cu), nickel (Ni), lead (Pb), aluminum (Al), and cadmium (Cd) are particularly significant due to their widespread industrial use and persistence in the environment. Chronic exposure to these metals has been associated with various health issues, including neurotoxicity, carcinogenesis, oxidative stress, and immune system dysfunction [1,2,3].

### 1.2. MicroRNAs and Metal-Induced Toxicity

Recent studies have highlighted the role of microRNAs (miRNAs) in mediating cellular responses to heavy metal toxicity, particularly in regulating gene expression related to inflammation, apoptosis, and oxidative stress [4,5]. miRNAs are regulatory RNAs consisting of 19–22 nucleotides that control gene expression by binding to complementary sequences on target messenger RNAs (mRNAs), thereby suppressing translation or inducing degradation. A large body of evidence suggests their involvement in critical processes such as proliferation, differentiation, and apoptosis [6]. Environmental toxicants, including metals, pharmaceuticals, and organic pollutants, can modulate miRNA expression patterns depending on dose and cellular context [7].

### 1.3. The Case of Aluminum Exposure

Aluminum (Al), the third most abundant element in the Earth’s crust, makes up over 8% of the planet’s surface. Although not essential for human biology, it is widely used in daily life—found in water purification systems, food additives, antacids, cosmetics, and vaccines. Despite its broad use, Al’s neurotoxicity has raised increasing concern due to its links to synaptic dysfunction and neuronal apoptosis [8,9,10,11].

### 1.4. Heavy Metals in Welding Fumes

Welding is a widely utilized industrial technique for joining metal components and is increasingly adopted across various sectors due to its efficiency. However, the process generates fumes containing hazardous metal particles, posing serious occupational health risks. The composition of welding electrodes varies depending on the electrode type and alloy specification; for instance, it may include approximately 19% chromium, 12% nickel, and 3% molybdenum, along with 0.8% silicon and 0.8% manganese [12]. These fumes may also contain other metals such as lead, aluminum, titanium, iron, zinc, and cadmium. Multiple studies have demonstrated strong correlations between environmental metal concentrations and biomarkers in blood and urine. Welding fume exposure has been linked to various health issues, including asthma, bronchitis, pneumoconiosis, lung cancer, neurological disorders, and impaired kidney and reproductive function [13]. Understanding the molecular mechanisms underlying these toxic effects—particularly how metal particles modulate miRNA expression—requires larger-scale studies. During stainless steel welding, fumes enriched with nickel (Ni), manganese (Mn), iron (Fe), and both trivalent and hexavalent chromium (Cr^III^ and Cr^VI^) compounds are released in varying amounts. These metal ions, especially those with high reduction potential, are believed to induce oxidative stress responses in the respiratory system, often following inflammatory activation [14].

### 1.5. Specific miRNAs in Metal Toxicity

miR-19a-3p and miR-19b-3p, part of the miR-17-92 cluster, are involved in proliferation, differentiation, and apoptosis regulation. Their downregulation by Cr(VI) exposure has been associated with carcinogenic pathways through PTEN and AKT signaling disruption [15,16].

miR-24-3p contributes to DNA damage repair and modulates apoptotic pathways by targeting BIM, BRCA1, and p53, indicating its role in maintaining genomic stability [17,18]. While specific studies on the impact of Cr exposure on miR-24-3p expression are limited, it is well-established that exposure to Cr(VI) induces oxidative stress and DNA damage.

### 1.6. Other Relevant Metals: Ni, Pb, Cd, and Cu

Nickel (Ni), lead (Pb), cadmium (Cd), and copper (Cu) are also toxic metals with distinct mechanisms. Ni exposure occurs mainly via inhalation and varies in toxicity based on chemical form [19]. Pb disrupts gene expression through epigenetic alterations such as DNA methylation and miRNA dysregulation, offering a potential biomarker for chronic toxicity [20]. Cd exposure leads to kidney and lung damage and also modulates miRNA expression to promote autophagy and oxidative stress responses [21,22,23,24]. Cu is essential for biological functions, but its dysregulation has been linked to neurodegenerative disorders such as Alzheimer’s disease [25,26].

### 1.7. Bioinformatics Approach and Study Aim

In this study, we used bioinformatics tools to identify miRNAs associated with genes responsive to Cr, Ni, Cu, Pb, and Al exposure. Databases such as CTD “https://ctdbase.org/ (accessed on 6 March 2025)” and Venn diagram generators from Biotools “https://www.biotools.fr/ (accessed on 6 March 2025)” were used to determine common target genes. miRNA–gene interaction networks were further explored using MIENTURNET. The study focuses on evaluating the expression of hsa-miR-19a-3p, hsa-miR-19b-3p, hsa-miR-130b-3p, hsa-miR-25-3p, hsa-miR-363-3p, hsa-miR-92a-3p, and hsa-miR-24-3p, using hsa-miR-16-5p and RNU6 (U6 snRNA) as reference genes.

## 2. Materials and Methods

### 2.1. Identification of Cr-, Ni-, Cu-, Pb-, and Al-Related Genes Through a Comparative Toxicogenomics Database (CTD)

The Comparative Toxicogenomics Database (CTD) (https://ctdbase.org/, accessed on 26 March 2025) was used to find genes linked to Cr, Ni, Cu, Pb, and Al. The CTD is a public database that aims to advance understanding of how environmental exposures affect human health. Chemical gene/protein interactions provide manually curated information on chemical–disease and gene–disease associations. These data are integrated with functional and pathway data to help develop hypotheses about the mechanisms underlying environmentally influenced diseases.

**Figure 1:**
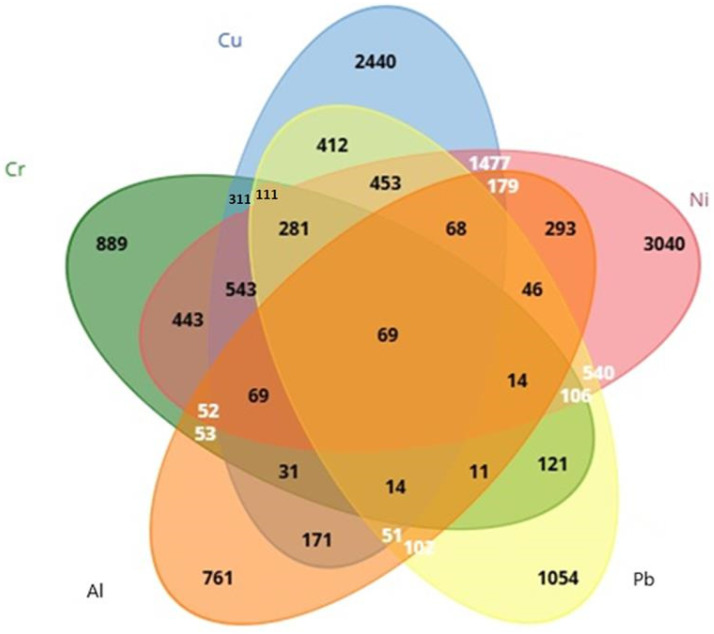
Evaluation of genes associated with Cr, Ni, Cu, Pb, and Al using a Venn diagram.

After CTD screening, it was found that 761 genes were linked to Al, 889 genes were linked to Cr, 2440 genes were linked to Cu, 3040 genes were linked to Ni, and 1054 genes were linked to Pb. Using Venn diagrams via the Biotools (https://www.biotools.fr/, accessed on 26 March 2025) web portal, 69 (ALB, BAX, C3, CASP3, CASP9, CAT, CXCL8, CYP7A1, FOS, GPX1, GSR, HAVCR1, HMOX1, IGF1, IGF2, IL1B, IL4, IL6, JUN, MAPK3, MAPT, MMP2, NFE2L2, PIK3R1, RELA, SLC2A1, SOD1, SOD2, TF, TGFB1, TNF, ACHE, ACSL4, ADH1B, ATF2, BCL2, BCL2L1, CASP12, COL1A1, CRP, CYCS, ELN, ESR1, FDFT1, FDPS, G6PD, GCHFR, IL2, KRT5, LRIG3, LYZ, MAP1LC3B, MCM8, MYC, NAA15, NOS2, PADI2, PINK1, PRKN, PTGS2, PTK2, RPL37, RRM2, SQSTM1, STAT1, TFRC, VEGFA, VIM, XDH69) common genes were detected from heavy metals: Cr, Cu, Ni, Pb, and Al (Figure 1).

### 2.2. MIENTURNET (MicroRNA ENrichment TURned NETwork) Analysis

In the present study, MIENTURNET’s network analysis was used to evaluate the relationships between target genes and miRNAs. The MIENTURNET online tool “http://userver.bio.uniroma1.it/apps/mienturnet/ (accessed on 6 March 2025)” was used to determine which miRNAs were induced by Cr, Ni, Cu, Pb, and Al based on genes associated with heavy metal exposure. We determined the biological significance of these miRNAs by constructing a miRNA target interaction network and performing functional enrichment analysis. miRNAs thought to play a role in Cr, Ni, Cu, Pb, and Al toxicity responses associated with 69 genes were investigated as a result of miRTarBase and TargetScan miRNA–target enrichment analyses.

The threshold value for the minimum number of miRNA–target interactions was taken as 1 and the threshold value for the adjusted *p* value (FDR) was taken as 1. From the analysis results obtained, hsa-miR-19a-3p, hsa-miR-130b-3p, hsa-miR-25-3p, hsa-miR-363-3p, hsa-miR-92a-3p, hsa-miR-24-3p, and hsa-miR-19b-3p miRNAs associated with Cr, Ni, Cu, Pb, and Al toxicity were included in the study. Two reference genes, hsa-miR-16-5p and RNU6 (U6 snRNA), were evaluated for normalization. Both were used to calculate ΔCt and ΔΔCt values for comparative analysis. The results based on U6 are presented in the Supplementary Materials.

The false discovery rate (FDR) was calculated using the nominal *p* value obtained from the hypergeometric test. We determined fold enrichment by dividing the genes in a pathway in the list by the background percentage. FDRs tend to be smaller for larger pathways due to increased statistical power. We use this measure to show how strongly a pathway overrepresents certain genes. Using the MIENTURNET miRNA–target enrichment analysis, the best pathways based on FDR were chosen from 69 genes linked to the toxicity of five heavy metals that were part of the study. The pathways were then ranked by fold enrichment, and the best miRNAs were found using miRTarBase (Table 1) and TargetScan (Table 2).

Accordingly, hsa-miR-19a-3p was found to have high interactions with ESR1 (Estrogen Receptor 1), TNF (tumor necrosis factor), TF (Transferrin), MYC (MYC Proto-Oncogene, BHLH Transcription Factor), ACSL4 (Acyl-CoA Synthetase Long Chain Family Member 4), and PRKN (Parkin RBR E3 Ubiquitin Protein Ligase) genes; hsa-miR-130b-3p with ESR1, ACSL4, MMP2 (Matrix Metallopeptidase 2), and IGF1 (Insulin Like Growth Factor 1) genes; hsa-miR-25-3p with the MYC gene; hsa-miR-363-3p with the CASP3 (Caspase 3) gene; hsa-miR-92a-3p with CYP7A1 (Cytochrome P450 Family 7 Subfamily A Member 1), GCHFR (GTP Cyclohydrolase I Feedback Regulator), and MYC genes; hsa-miR-24-3p with MYC, TGFB1 (Transforming Growth Factor Beta 1), RRM2 (Ribonucleotide Reductase Regulatory Subunit M2), HMOX1 (heme oxygenase 1 gene), IGF1, IL4 (Interleukin-4), IL1B (Interleukin 1 Beta), and TNF genes; and hsa-miR-19b-3p with ESR1, LRIG3 (Leucine Rich Repeats And Immunoglobulin Like Domains 3), ATF2 (Activating Transcription Factor 2), TGFB1, ACSL4, and PRKN genes via miRTarBase database (Table 1).

In the TargetScan database, it was determined that hsa-miR-19a-3p/hsa-miR-19b-3p had high interaction with ESR1, LRIG3, ATF2, IGF1, ACSL4, HAVCR1 (Hepatitis A Virus Cellular Receptor 1), and SLC2A1 (solute carrier family 2 member 1) genes; hsa-miR-130a-3p with IGF1, ESR1, ACSL4, SLC2A1, and TNF genes; hsa-miR-25-3p/hsa-miR-363-3p/hsa-miR-92a-3p with the NAA15 (N-Alpha-Acetyltransferase 15, NatA Auxiliary Subunit) gene; and hsa-miR-24-3p with the G6PD (Glucose-6-Phosphate Dehydrogenase) gene (Table 2).

### 2.3. Sample Size

In the study, sample size calculation was performed using the ANOVA method, and it was calculated using an open access website as the software (https://www.statskingdom.com/sample_size_regression.html accessed on 27 March 2025). In the calculation method (in miRNA values measured according to the reference miRNA value, in any group with an estimated standard deviation of 1 units or a 1-unit difference (Type I error 0.05, Type II error 0.20, power 0.80)), the minimum sample size for each group is 16 people. Blood samples were analyzed by taking them from a company that has an occupational physician and regularly follows occupational safety measures and that performs aluminum casting and welding operations. Office workers without any diagnosed diseases and without exposure to aluminum and welding fumes made up the control group.

The Public Health Department of the Istanbul Faculty of Medicine evaluated workers who applied to the Occupational Diseases Polyclinic for diagnostic accuracy, took their consent forms and blood samples, and began the study. The study included 16 workers between the ages of 20–55 who were exposed to aluminum and had no disease, 16 workers who were exposed to welding fumes and had no disease, and 16 healthy controls who were known not to have been exposed to aluminum or welding fumes. Istanbul University, Faculty of Science, Department of Biology University Trace Element Analysis Laboratory tested serum samples taken from workers and healthy controls for aluminum and welding fume exposure. The inductively coupled plasma–optical emission spectrometry (ICP-OES) method was used to find out how much of the heavy metals Al, Cd, Cr, Cu, Ni, and Pb were in serum samples. The methodological study aims to identify miRNAs potentially associated with heavy metal exposure.

The age range of workers exposed to aluminum was 20–55. The age range of workers exposed to welding fumes was 20–54. The age range of healthy control groups was 32–55. All volunteers participating in the study were male. The Istanbul Medical Faculty Clinical Research Ethic Committee approved our research on January 8, 2025, with the following number and date: E-29624016-050.99-3104384. Each individual gave their express consent before taking part.

### 2.4. Serum Preparation

A measure of 10 mL of venous blood was placed in vacuum-dry blood collection tubes and incubated at room temperature for 30 min. Then, it was first centrifuged at 4 °C at 1900× *g* for 10 min, and then the upper serum phase was transferred to a new tube and then centrifuged again at 4 °C at 3000× *g* for 15 min. The completed serum was separated into Eppendorf tubes and frozen at −80 °C for later use.

### 2.5. Microwave Acid Extraction

In order to use ICP-OES (inductively coupled plasma–optical emission spectrometry) to measure heavy metal levels, serum samples must first be broken down and turned into a solution. First, we used 1 mL of serum samples. The serum sample was put in Teflon containers of a Berghoff wet separation device (Italy) along with 1.9 mL of nitric acid, 2 mL of hydrogen peroxide, and 3 mL of ultrapure water. The samples were heated in a microwave to 130 °C for 8 min, then 155 °C for 5 min with 5 °C increases, 185 °C for 12 min with 5 °C increases, 100 °C for 5 min with 5 °C increases, and finally 50 °C for 5 min with a sudden drop in pressure. Acid extraction was then performed on the samples.

### 2.6. ICP-OES Parameters

Quantitative analyses of heavy metals for serum samples were performed using a PerkinElmer brand Optima 7000DV model ICP-OES device (PerkinElmer, Connecticut, US) with a concentric nebulizer, standard-section cyclonic spray chamber, alumina injector, and quartz torch. We accepted the relative standard deviation as 10% and carried out nine replicate analyses. We tested the device’s sensitivity and accuracy with heavy metal mixtures of 0.6, 0.8, 1.0, 2.0, 4.0, and 5.0 μg/L and an internal standard of 1.0 μg/L manganese. Before analyzing real samples, we performed calibration and determined stability. It was set so that the RF power was 1.3 kW, the nebulizer gas flow rate was 0.6 (L·min^−1^), the auxiliary gas flow rate was 0.2 (L·min^−1^), the plasma gas flow rate was 16.0 (L·min^−1^), the sample aspiration rate was 1.0 (mL·min^−1^), the platinum element detection wavelengths were 265.945 and 214.423 nm, the argon flow rate was 8 bar, and the nitrogen flow rate was 5 bar.

### 2.7. RNA Isolation and cDNA Synthesis

miRNAs were isolated from patients’ serum samples and control serum samples using the miRNeasy Serum/Plasma Kit (catalog no. 217204; Qiagen, Germantown, MD, USA), according to the manufacturer’s instructions. In order to determine the amount and quality of RNA, the NanoDrop 2000c (Thermo Fisher Scientific, Waltham, MA, USA) was used (A260/A280: 1.8–2.0). cDNA synthesis from samples was performed using miRCURY LNA RT Kit (catalog no. 339340; Qiagen, Germantown, MD, USA) and Applied Biosystems 2720 Thermal Cycler (Tustin, CA, USA). The expression levels of miRNAs were determined using the miRCURY LNA SYBR Green PCR Kit (catalog no. 339346; Qiagen, USA) and the QIAGEN Rotor-Gene Q. The miRNA assay information used for miRCURY LNA miRNA PCR analysis is given in Table 3.

### 2.8. Quantitative Real-Time Polymerase Chain Reaction (qRT-PCR)

The Rotor-Gene Q RT-PCR device (Qiagen, USA) received a total reaction volume of 10 μL from the Qiagen RT2 SYBR Green qPCR mastermix kit, which included 5 μL of 2× miRCURY SYBR green master mix, 1 μL of ddH2O, 1 μL of resuspended PCR primer mix, and 3 μL of cDNA template (diluted 1:30) (Table 4). hsa-miR 16-5p was selected as the reference gene. The PCR process was performed once.

### 2.9. Data Analysis and Statistics

The variables obtained from the study at the proportional measurement level are presented as mean, median, standard deviation, minimum, and maximum, and variables at the classificatory measurement level are presented as number and percentage. The distribution of age, working years, and smoking among the three groups and heavy metal analysis results was investigated using a one-way analysis of variance (ANOVA). Post hoc comparisons were conducted using Tukey’s HSD test. Additionally, Dunnett’s T3 test was applied due to unequal variances as a sensitivity analysis.

Using Ct data obtained from RT-PCR experiments, fold changes were calculated for each miRNA relative to each reference miRNA in the examined condition using the Livak (2^−ΔΔCt^) method. Statistical evaluation was performed using ΔCt values calculated with hsa-miR-16-5p as the reference miRNA across aluminum-exposed, source-exposed, and control groups. In addition, parallel analyses utilizing U6 snRNA for normalization are provided in the Supplementary Materials, including comparative assessments of significance across the two reference genes.

hsa-miR-16-5p has been utilized as a reference gene in various qRT-PCR studies due to its stable expression across different conditions. However, its suitability as a reference gene in metal exposure studies requires careful validation, as metal exposure may influence its expression levels.

A comparison of different miRNA values was performed using the Mann–Whitney U test according to the study year group and smoking group. Two-way analysis of variance was used to compare the interaction of each miRNA difference value with the smoking year group and study year group.

In terms of occupational health and safety, it is very important to know how accurately individuals exposed to heavy metals can be distinguished from healthy individuals. In the decision-making process, ROC (Receiver Operating Characteristic) analysis was used to assess the discriminatory power of the test. We conducted the ROC analysis using the licensed software IBM SPSS 30 for Windows, provided by Istanbul University “https://bilgiislem.istanbul.edu.tr/tr/yalis/Veri%20Analizi/ (accessed on 6 March 2025)”. Statistical significance was determined using a two-tailed test, with *p* < 0.05 considered significant.

## 3. Results

### 3.1. Demographic Information Distributions

When the ages, working years, and smoking distribution of the participants were examined, no statistically significant difference was found (*p* > 0.05) (Table 5).

### 3.2. Serum Heavy Metal Analyses

The simultaneous biological monitoring of chromium, cadmium, aluminum, nickel, copper, and lead in the serum of aluminum workers, welding workers, and a control group was performed using ICP-OES. The results of the metal concentrations measured in the serum of aluminum workers, welding workers, and the control group are presented in Table 6.

The results shown in Table 4 present the serum concentrations tested in aluminum workers, welding workers, and controls. The mean serum concentrations of metal workers were significantly higher in Al and Pb compared to the control group. These differences were found to be statistically significant (*p* < 0.001). Although Cu, Cr, and Ni concentrations were higher in metal and aluminum workers, the differences were not statistically significant (*p* > 0.05).

### 3.3. Correlation Analysis

When Table 7 is examined, weak positive correlations are observed between serum aluminum values and hsa-miR-19a-3p (r = 0.319, *p* = 0.027), hsa-miR-19b-3p (r = 0.413, *p* = 0.003), hsa-miR-130b-3p (r = 0.356, *p* = 0.013), hsa-miR-25-3p (r = 0.304, *p* = 0.036), hsa-miR-92a-3p (r = 0.354, *p* = 0.014), and hsa-miR-24-3p (r = 0.411, *p* = 0.004). Serum chromium values showed weak positive correlations with hsa-miR-363-3p (r = 0.383, *p* = 0.03) and hsa-miR-92a-3p (r = 0.363, *p* = 0.041). Additionally, a weak negative correlation was observed between serum copper values and hsa-miR-363-3p (r = 0.295, *p* = 0.042).

We found no statistically significant relationship between serum nickel levels and the ΔCt values of all miRNAs (*p* > 0.05). However, weak positive correlations were observed between serum lead levels and the miRNA values of hsa-miR-130b-3p, hsa-miR-92a-3p, and hsa-miR-24-3p, with correlation coefficients of r = 0.399, *p* = 0.005; r = 0.370, *p* = 0.01; and r = 0.478, *p* < 0.001, respectively.

### 3.4. Differential microRNA Expression Analyses

When comparing the 16 aluminum-exposed participants and control groups, the ΔCt values for the housekeeping gene hsa-miR-16-5p showed that hsa-miR-19a-3p and hsa-miR-19b-3p exhibited statistically significant differences (*p* < 0.05). However, the expression levels of hsa-miR-130b-3p, hsa-miR-25-3p, hsa-miR-363-3p, hsa-miR-92a-3p, and hsa-miR-24-3p did not differ significantly between the two groups (*p* > 0.05). Additionally, hsa-miR-16-5p indicated that the ΔCt values for hsa-miR-19a-3p, hsa-miR-130b-3p, hsa-miR-92a-3p, and hsa-miR-24-3p were statistically significant (*p* < 0.05) between the 16 welding fume-exposed participants and control groups (Table 8). Parallel results using U6 normalization are available in the Supplementary Materials, including comparisons of significance across reference genes.

When comparing aluminum-exposed individuals with non-aluminum-exposed individuals, hsa-miR-19a-3p, hsa-miR-19b-3p, hsa-miR-130b-3p, hsa-miR-25-3p, hsa-miR-363-3p, hsa-miR-92a-3p, and hsa-miR-24-3p showed downregulation, with fold changes of −1.58, −1.69, −1.53, −1.33, −1.01, −1.16, and −1.49, respectively. Similarly, when comparing individuals exposed to welding fumes with those not exposed, hsa-miR-19a-3p, hsa-miR-19b-3p, hsa-miR-130b-3p, hsa-miR-25-3p, hsa-miR-363-3p, hsa-miR-92a-3p, and hsa-miR-24-3p were downregulated, with fold changes of −1.35, −1.16, −2.36, −1.06, −1.24, −1.76, and −2.46, respectively (Table 8).

### 3.5. Two-Way Analysis of Variance (ANOVA)

We evaluated how two independent variables (study year and smoking) affected a dependent variable (miRNA). We tested the effect of more than one independent variable on the dependent variable at the same time and examined whether there was an interaction between the independent variables. The miRNAs hsa-miR-19a-3p, hsa-miR-19b-3p, hsa-miR-130b-3p, hsa-miR-92a-3p, and hsa-miR-24-3p, which were found to differ between the groups, were evaluated with a two-way analysis of variance based on study year and smoking status.

The ΔCt values for hsa-miR-19a-3p, hsa-miR-130b-3p, hsa-miR-25-3p, hsa-miR-363-3p, hsa-miR-92a-3p, hsa-miR-24-3p, and hsa-miR-19b-3p miRNAs, obtained according to the study groups and the hsa-miR-16-5p reference gene, are provided in Table 8, Table 9, Table 10, Table 11, Table 12 and Table 13, based on study year and smoking status. The years of work revealed a significant difference in the hsa-miR-24-3p Ct value of the control group. This difference was significantly higher compared to those who had worked for 11 years or more (Table 9).

The years of study revealed no significant difference in the ΔCt values of the aluminum group (*p* > 0.05) (Table 10).

Depending on their years of experience. welding workers showed a significant difference in their hsa-miR-363-3p Ct values. The difference was significantly greater for workers with 11 or more years of experience (*p* < 0.05) (Table 11).

We found no statistically significant correlation between Ct values and the number of years of smoking in the control group (Table 12).

The aluminum workers group did not show statistically significant Ct values based on the number of years smoking (Table 13).

In the evaluations according to study year, a significant difference in ΔCt hsa-miR-24-3p was found in individuals who had worked for 11 years or more (*p* < 0.05). In welding workers, a significant difference was observed in ΔCt hsa-miR-363-3p in those who had worked for 11 years or more (*p* < 0.05). The evaluations based on smoking status revealed a significant difference in ΔCt hsa-miR-19a-3p and ΔCt hsa-miR-25-3p among welding workers. Those who smoked one pack of cigarettes per day for more than 5 years showed a significantly higher difference in hsa-miR-19a-3p and hsa-miR-25-3p (*p* < 0.05) (Table 14). It is suggested that metal fume exposure and smoking influence both hsa-miRs.

When the important ΔCt values were analyzed, several miRNAs showed differences between the source group and the control group based on both the study year and the number of years of smoking. However, in the source group, hsa-miR-24-3p exhibited a two-way interaction. In the source group, the trend was not in the same direction as in the control and aluminum groups. No significant differences were found in other miRNAs between the study group, study year, and smoking group. We observed that the two-way interaction followed the same direction. The F values and *p*-values obtained indicate whether the effects of the independent variables on the dependent variable are significant. Table 15, Table 16, Table 17, Table 18, Table 19, Table 20, Table 21, Table 22, Table 23, Table 24, Table 25 and Table 26 present the results of our examination of the interaction effect between the independent variables.

In ΔCt hsa-miR-19a-3p, the group and study year interaction (F = 0.272; *p* = 0.763) was not found to be statistically significant (Table 15).

In ΔCt hsa-miR-19a-3p, the group and smoking year interaction (F = 0.483; *p* = 0.62) was not found to be statistically significant (Table 16).

In ΔCt hsa-miR-19b-3p, the group and study year interaction (F = 0.46; *p* = 0.634) was not found to be statistically significant (Table 17).

In ΔCt hsa-miR-19b-3p, the group and smoking year interaction (F = 1.109; *p* = 0.339) was not found to be statistically significant (Table 18).

In ΔCt hsa-miR-24-3p, the group and study year interaction (F = 3.428; *p* = 0.042) was found to be statistically significant (Table 19).

In ΔCt hsa-miR-24-3p, the group and smoking year interaction (F = 1.197; *p* = 0.312) was not found to be statistically significant (Table 20).

In ΔCt hsa-miR-92a-3p, the group and study year interaction (F = 0.078; *p* = 0.925) was not found to be statistically significant (Table 21).

In ΔCt hsa-miR-92a-3p, the group and smoking year interaction (F = 2.414; *p* = 0.102) was not found to be statistically significant (Table 22).

ΔCt hsa-miR-363-3p group and study year interaction (F = 0.273; *p* = 0.762) was not found to be statistically significant (Table 23).

ΔCt hsa-miR-363-3p group and smoking year interaction (F = 0.26; *p* = 0.772) was not found to be statistically significant (Table 24).

ΔCt hsa-miR-130b-3p group and study year interaction (F = 0.454; *p* = 0.638 was not found to be statistically significant (Table 25).

ΔCt hsa-miR-130b-3p group and smoking year interaction (F = 0.896; *p* = 0.416) was not found to be statistically significant (Table 26).

**Figure 2:**
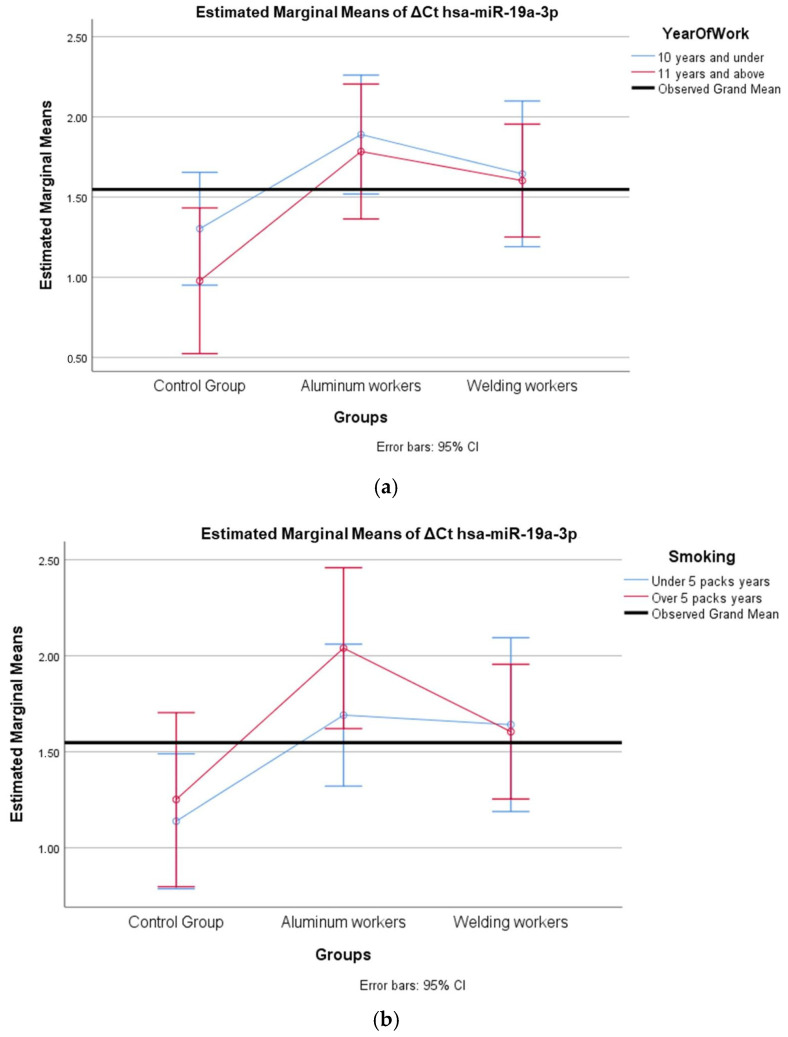
(**a**) Two-way interaction in hsa-miR-19a-3p by group and study year (F = 0.27; *p* = 0.76). (**b**) Two-way interaction in hsa-miR-19a-3p by group and smoking year (F = 0.48; *p* = 0.82).

**Figure 3:**
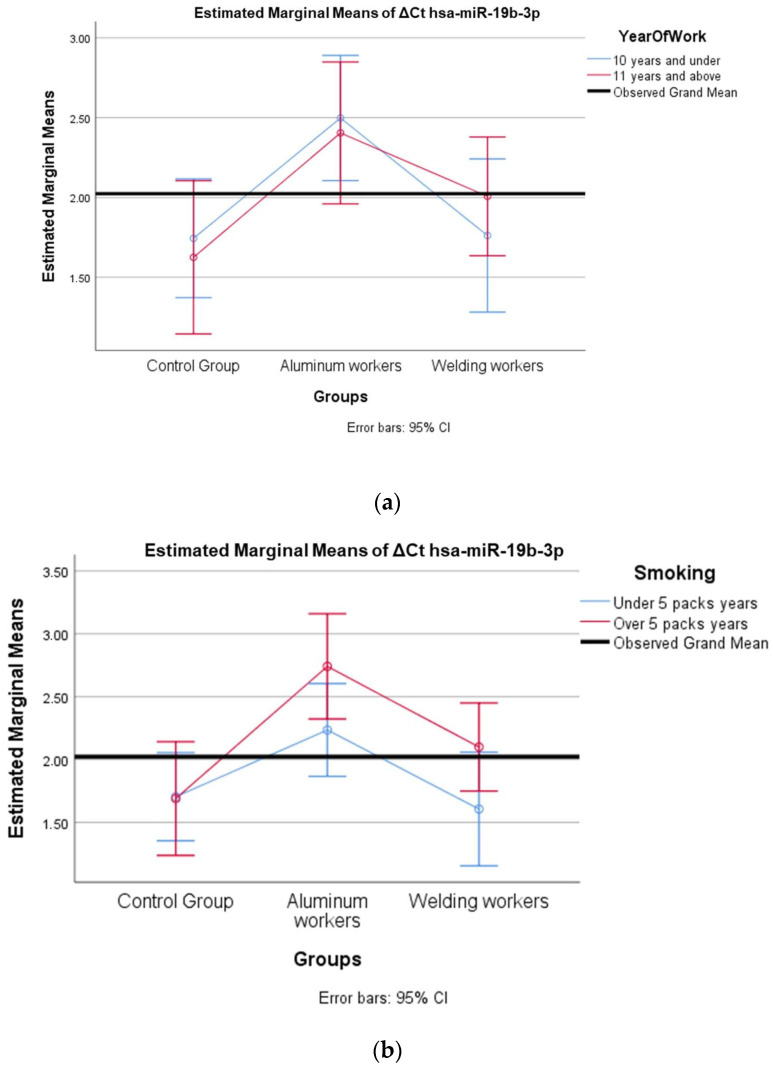
(**a**) Two-way interaction in hsa-miR-19b-3p by group and study year (F = 0.46; *p* = 0.63). (**b**) Two-way interaction in hsa-miR-19b-3p by group and smoking year (F = 1.18; *p* = 0.33).

**Figure 4:**
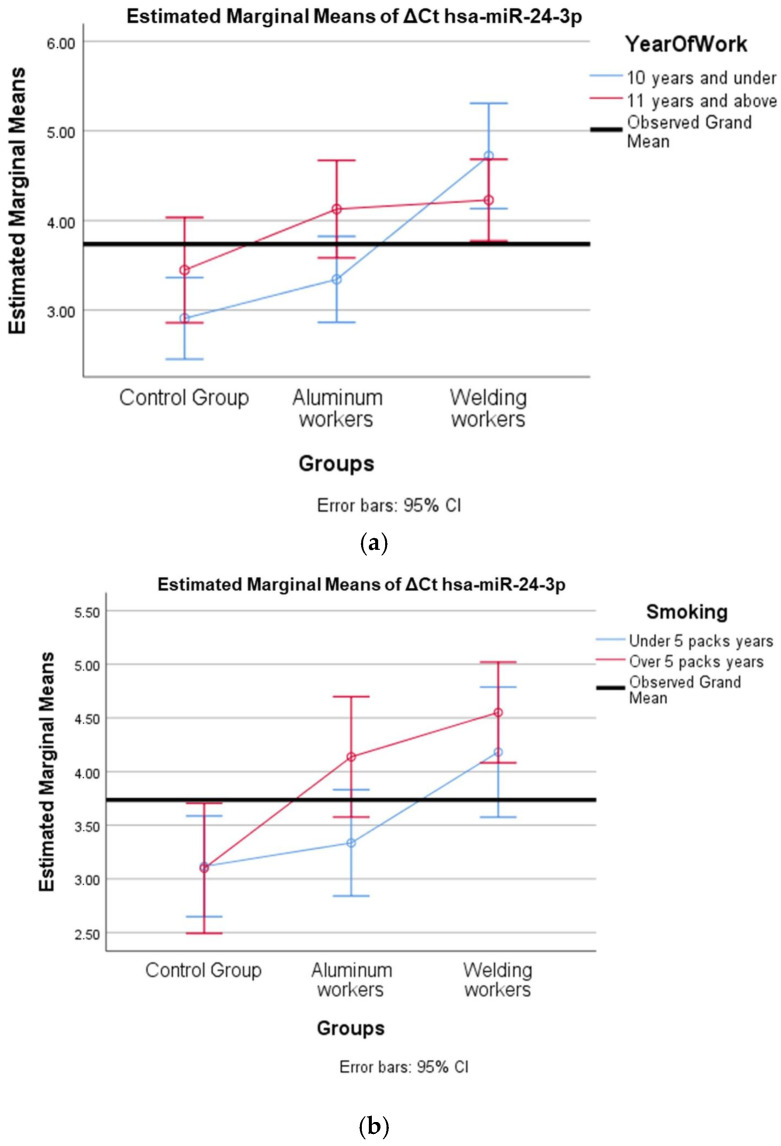
(**a**) Two-way interaction in hsa-miR-24-3p by group and study year (F = 3.42; *p* = 0.04). (**b**) Two-way interaction in hsa-miR-24-3p by group and smoking year (F = 1.19; *p* = 0.31).

**Figure 5:**
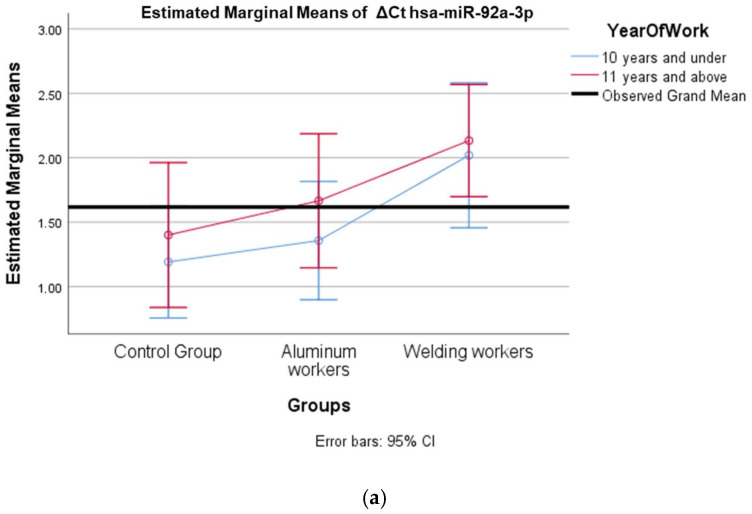

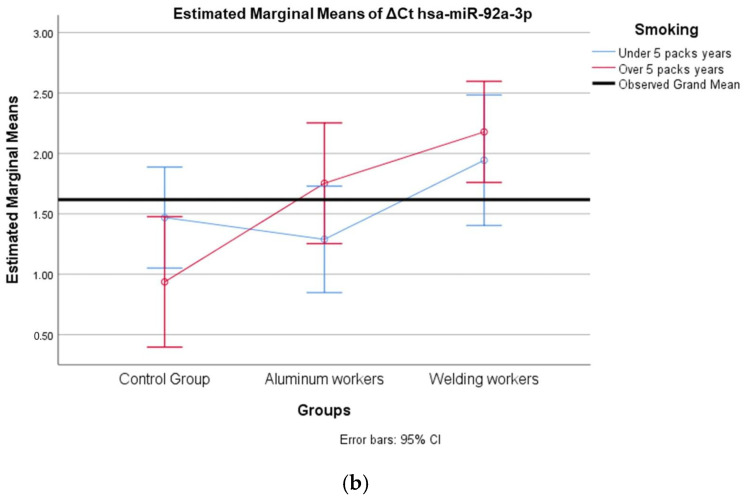
(**a**) Two-way interaction in hsa-miR-92a-3p group by group and study year (F = 0.07; *p* = 0.92). (**b**) Two-way interaction in hsa-miR-92a 3p by and smoking year (F = 2.41; *p* = 0.10).

**Figure 6:**
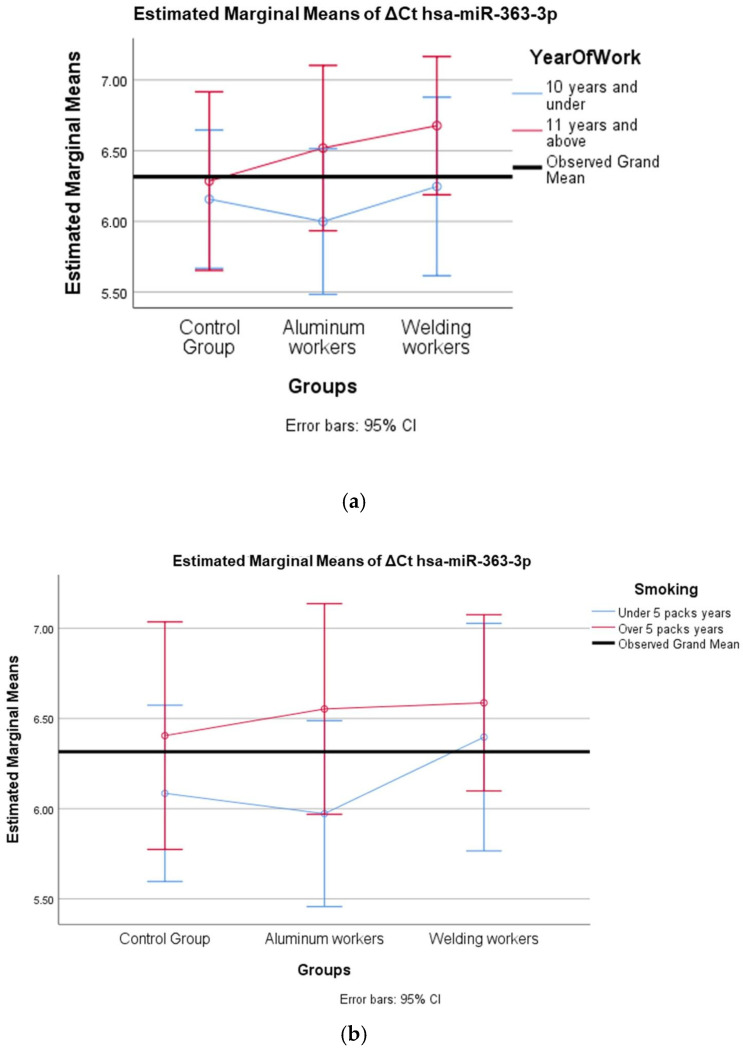
(**a**) Two-way interaction in hsa-miR-363-3p according to group and study year (F = 0.27; *p* = 0.78). (**b**) Two-way interaction in hsa-miR-363-3p according to group and smoking year (F = 0.26; *p* = 0.77).

The values of hsa-miR-19a-3p, hsa-miR-19b-3p, hsa-miR-24-3p, hsa-miR-363-3p, and hsa-miR-92a-3p, which were found to be significant among the three groups, were evaluated using a two-way analysis of variance based on working years and smoking status. The results are presented in Figure 2a,b, Figure 3a,b, Figure 4a,b, Figure 5a,b and Figure 6a,b.

The interaction of ∆Ct values for six of the seven hsa-miRs included in the study was analyzed in relation to the study year and smoking year (Table 15, Table 16, Table 17, Table 18, Table 19, Table 20, Table 21, Table 22, Table 23, Table 24, Table 25 and Table 26). A two-way interaction was found only in ΔCt hsa-miR-24-3p between the working year and groups. The ΔCt hsa-miR-24-3p value in welding workers differs according to the working years (Table 19). The differences in ΔCt hsa-miR-24-3p values in welding workers are not consistent in direction. This result suggests that people are affected differently by welding fumes over time (F = 3.42; *p* = 0.04, Figure 4a). The interaction between the working groups and smoking years was not found to be statistically significant in all ΔCt miR values (*p* > 0.05).

### 3.6. ROC Analysis

In aluminum and welding workers, we identified five serum miRNAs (hsa-miR-19a-3p, hsa-miR-19b-3p, hsa-miR-130b-3p, hsa-miR-92a-3p, and hsa-miR-24-3p) as significant compared to the reference miRNA hsa-miR-16-5p. We applied ROC analysis to these five significant serum miRNAs to identify a discriminatory miRNA in aluminum and welding workers.

**Figure 7:**
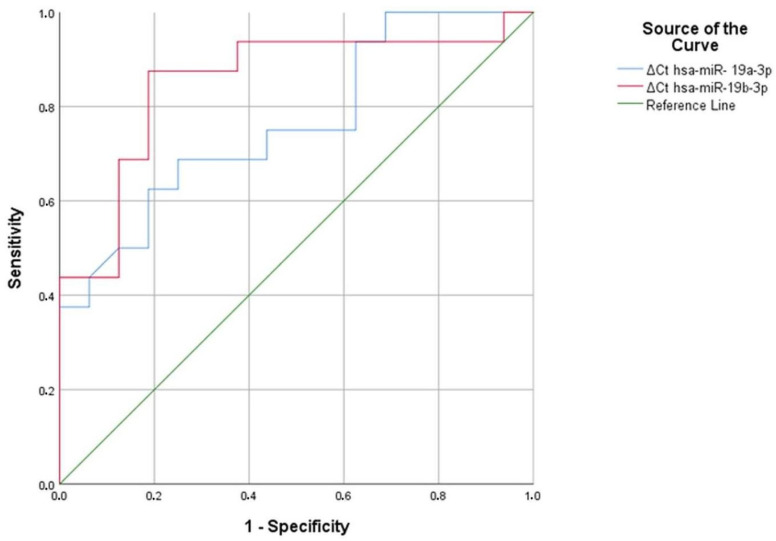
Significant miRNA values in aluminum workers compared to the control group.

As a result of the analysis, hsa-miR-19a-3p and hsa-miR-19b-3p were found to be significant in aluminum workers, with area under the curve (AUC) values of 0.764 and 0.852, respectively (*p* = 0.011, *p* = 0.001) (Figure 7) (Table 27). Similarly, in hsa-miR-19a-3p, a ∆Ct value above 1.47 at the positivity cut-off value can distinguish between individuals exposed to 69% aluminum and those not exposed to 75% aluminum. Similarly, in hsa-miR-19a-3p, a ∆Ct value above 1.59 at the positivity cut-off value can distinguish between individuals exposed to 69% aluminum and those not exposed to 75% aluminum. In hsa-miR-19b-3p, a ∆Ct value above 2.03 at the positivity cut-off value can distinguish between individuals exposed to 88% aluminum and those not exposed to 81% aluminum. In addition, in hsa-miR-19a-3p, a ∆Ct value above 2.08 at the positivity cut-off value can distinguish between individuals exposed to 75% aluminum and those not exposed to 81% aluminum (Table 28). According to ROC analysis, people exposed to aluminum can use hsa-miR-19a-3p and hsa-miR-19b-3p as markers.

**Figure 8:**
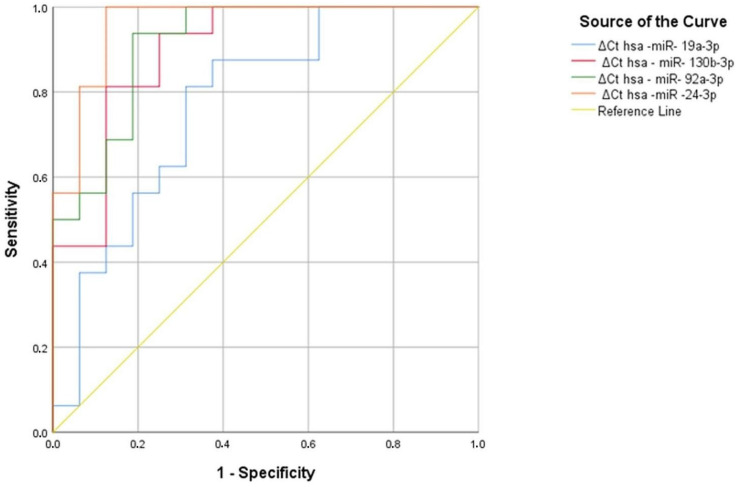
Significant miRNA values in welding workers compared to the control group.

As a result of the analysis, hsa-miR-130b-3p, hsa-miR-92a-3p, hsa-miR-24-3p, and hsa-miR-19a-3p were found to be significant in welding workers, with area under the curve (AUC) values of 0.773 (*p* = 0.008), 0.898 (*p* < 0.001), 0.914 (*p* < 0.001), and 0.961 (*p* < 0.001), respectively (Figure 8, Table 29).

A hsa miR-19a-3p Ct value greater than 1.39 has the ability to discriminate between 81% of the welding workers and 69% of the controls. The hsa-miR-130-3p ΔCt value of 1.44 has the power to discriminate between 75% of the source workers and 69% of the controls. A hsa-miR-130-3p Ct value greater than 7.61 has the ability to discriminate between 81% of the welding workers and 81% of the controls. The hsa-miR-130-3p ΔCt value of 7.67 has the power to discriminate between 81% of the source workers and 88% of the controls. The hsa-miR-92a-3p ΔCt value of 1.57 has the power to discriminate between 94% of the welding workers and 75% of the controls. The Hsa-miR-92a-3p ΔCt value of 1.83 has the power to discriminate 94% of the source workers and 81% of the controls. In hsa-miR-24-3p, a ∆Ct value above 6.39 at the positivity cutoff value tells the difference between people who were exposed to the welding by 69% and people who were not exposed to the welding by 75%. In hsa-miR-24-3p, a ∆Ct value above 6.46 at the positivity cutoff value tells the difference between people who were exposed to the welding by 69% and people who were not exposed to the welding by 75%. (Table 30). As a result of ROC analysis, it can be said that hsa-miR-19a-3p, hsa-miR-130b-3p, hsa-miR-92a-3p, and hsa-miR-24-3p can be used as a marker in individuals exposed to welding workers.

### 3.7. Independent Cohort Validation via GEO Database

We selected the GEO dataset “GSE63087” from the GEO database “https://www.ncbi.nlm.nih.gov/geo/ (accessed on 6 March 2025)” for the independent cohort validation of our study. In the study titled “Expression of viral and human microRNAs in blood in The Beijing Truck Driver Air Pollution Study (BTDAS)”, a total of 240 peripheral blood samples were taken from 120 participants—60 truck drivers, and 60 office workers—on both examination days, and the NanoString nCounter Human miRNA assay method was applied.

**Figure 9:**
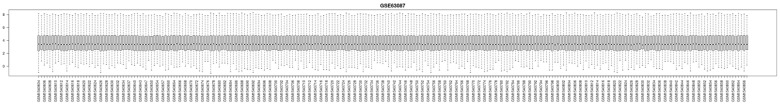
Distribution of values for selected samples as a boxplot.

In the *p* value calculation, the Benjamini and Hochberg (False Discovery Rate) method was used to compare two sample groups to determine differentially expressed miRNAs via R 4.2.2, Biobase 2.58.0, GEOquery 2.66.0, and limma 3.54.0 version. We found that the GSE63087 raw dataset had hsa-miR-19a, hsa-miR-130b, hsa-miR-25, hsa-miR-363, hsa-miR-92a, hsa-miR-24, and hsa-miR-19b, which are all miRNAs that were part of our study. We display the distribution of values from selected samples below (Figure 9).

## 4. Discussion

### 4.1. Key miRNAs Associated with Heavy Metal Exposure

The identification of specific miRNAs, including hsa-miR-19a-3p, hsa-miR-130b-3p, hsa-miR-25-3p, hsa-miR-363-3p, hsa-miR-92a-3p, hsa-miR-24-3p, and hsa-miR-19b-3p, is crucial for understanding the molecular responses to heavy metal exposure. These miRNAs play significant roles in regulating oxidative stress, apoptosis, inflammation, and DNA damage repair, which are key mechanisms of metal-induced toxicity [1]. In our study, qRT-PCR analysis revealed that hsa-miR-19a-3p and hsa-miR-19b-3p were significantly downregulated in individuals exposed to aluminum, as confirmed by ROC curve analysis. Additionally, four miRNAs—hsa-miR-19a-3p, hsa-miR-130b-3p, hsa-miR-92a-3p, and hsa-miR-24-3p—were significantly associated with welding fume exposure. These miRNAs demonstrated diagnostic potential, while the others showed limited discriminative power.

### 4.2. Expression Trends and Diagnostic Potential

We observed that the expression levels of seven miRNAs obtained from serum were decreased (down-regulated) in both aluminum-exposed and welding fume-exposed workers. Our study suggests that hsa-miR-19a-3p may be an important biomarker for both aluminum exposure (*p* = 0.009) and welding fume exposure (*p* = 0.010).

### 4.3. Target Gene Interactions and Pathway Involvement

MiRNA–target enrichment analysis of hsa-miR-19a-3p, hsa-miR-19b-3p, and hsa-miR-363-3p, which were found to be significant in aluminum-exposed workers, revealed strong interactions of hsa-miR-19a-3p and hsa-miR-363-3p with the *ESR1*, *TNF*, *TF*, *MYC*, *ACSL4*, *PRKN*, and *CASP3* genes. Additionally, hsa-miR-19b-3p, which was identified as significant in workers exposed to both aluminum and welding fumes, exhibited high interaction with the *ESR1*, *LRIG3*, *ATF2*, *TGFB1*, *ACSL4*, and *PRKN* genes. Furthermore, hsa-miR-130b-3p, hsa-miR-25-3p, hsa-miR-92a-3p, and hsa-miR-24-3p, which were found to be significant in workers exposed to welding fumes, showed strong interactions with multiple genes, including *ESR1*, *ACSL4*, *MMP2*, *IGF1*, *MYC*, *CYP7A1*, *GCHFR*, *TGFB1*, *RRM2*, *HMOX1*, *IGF1*, *IL4*, *IL1B*, and *TNF.*

### 4.4. Aluminum- and Chromium-Specific Observations

In our study, hsa-miR-19a-3p and hsa-miR-19b-3p were significantly downregulated in aluminum-exposed workers (*p* < 0.05). Weak positive correlations were also observed between aluminum levels and the expression of hsa-miR-19a-3p, hsa-miR-19b-3p, hsa-miR-130b-3p, hsa-miR-25-3p, hsa-miR-92a-3p, and hsa-miR-24-3p (*p* < 0.05). Previous research indicates that exposure to aluminum, particularly in the form of aluminum maltolate, leads to the downregulation of miR-19a and miR-19b in human neuroblastoma SH-SY5Y cells [11]. This downregulation is associated with increased neural cell apoptosis, mediated by the modulation of apoptosis-related proteins such as PTEN, *p*-AKT, and p53. Inhibition of miR-19a or miR-19b alone reduces cell viability and activates caspase-9 and caspase-3, further promoting apoptosis. These findings suggest that maintaining the expression of these miRNAs is crucial for neuronal survival under aluminum exposure [11].

Oxidative stress has also been shown to downregulate miR-92a-3p. In a study by Li et al., oxidative stress-induced exosomes in endothelial cells reduced miR-92a-3p levels and promoted angiogenesis by affecting PTEN, p-AKT, and p53 signaling [27]. Moreover, decreased levels of miR-92a-3p have been observed in plasma samples of patients with mild cognitive impairment and Alzheimer’s disease, suggesting its role in synaptic dysfunction and neurodegeneration [28]. These observations support the relevance of miR-92a-3p in aluminum-induced neurotoxicity.

In the welding fume-exposed group, hsa-miR-19a-3p, hsa-miR-130b-3p, hsa-miR-92a-3p, and hsa-miR-24-3p were also found to be downregulated (*p* < 0.05). Weak but significant positive correlations were found between chromium exposure and hsa-miR-363-3p and hsa-miR-92a-3p (r = 0.383 and 0.363, respectively; *p* < 0.05). Both miR-19a-3p and miR-19b-3p, members of the miR-17-92 cluster, are known to regulate PTEN, a tumor suppressor gene within the AKT pathway. Exposure to hexavalent chromium [Cr(VI)] has been shown to downregulate these miRNAs, leading to enhanced AKT activation and suppressed apoptosis, which may increase carcinogenic potential [29].

Recent studies further support the downregulation of miR-19a-3p in Cr(VI)-exposed workers, often accompanied by decreased levels of miR-19b-3p and miR-142-3p, and upregulation of miR-590-3p and miR-941 [30]. Although miR-590-3p was not among the miRNAs directly analyzed in this study, its biological relevance in cellular stress contexts is noteworthy. Overexpression of miR-590-3p has been shown to support cellular defense mechanisms in specific conditions. For instance, in cardiomyocytes, it inhibits apoptosis and autophagy under hypoxia/reperfusion stress by targeting HIF-1α, thereby promoting cell survival. These findings suggest that miR-590-3p may act as a potential therapeutic target in myocardial ischemia/reperfusion injury [31]. However, its biological effects appear to be highly context-dependent. In several types of cancer, including hepatocellular carcinoma and colorectal cancer, miR-590-3p has been associated with oncogenic activity. It encourages cells to grow by affecting tumor suppressor genes like MDM2 and reduces the activity of Hippo pathway components such as LATS1 and SAV1, which are important for controlling cell growth and programmed cell death [32,33]. These dual effects underscore the importance of evaluating miR-590-3p expression and function in a tissue-specific and disease-specific manner. While it may play a protective role in certain physiological responses, its dysregulation could also contribute to pathogenesis under different biological conditions. Therefore, any future therapeutic approach involving miR-590-3p should carefully consider dosage, duration, cellular context, and potential off-target effects.

miR-19a-3p plays a role in endothelial function, and its overexpression has been shown to protect against atherosclerosis-associated endothelial dysfunction. Therefore, its downregulation due to chromium exposure could impair vascular function and may serve as a biomarker for exposure and associated risks [34]. So, we can say that chromium exposure is linked to miR-19a-3p downregulation, which could affect the health of blood vessels and be used as a biomarker to measure exposure.

Hsa-miR-24-3p is involved in regulating cellular stress responses, including DNA damage repair and apoptosis. Its decreased expression has been associated with enhanced susceptibility to apoptosis and emphysema, particularly in the context of cigarette smoke exposure [17]. Considering that Cr(VI) is a known human carcinogen associated with lung cancer and oxidative stress [35], it is plausible that chromium may also influence miR-24-3p expression. However, more research is needed to confirm this relationship.

Our study also found weak negative correlations between copper levels and hsa-miR-363-3p expression (r = –0.295; *p* = 0.042). Although no literature directly links copper exposure to miR-363-3p, this miRNA is known to regulate inflammation, oxidative stress, and apoptosis. It has been shown to reduce endothelial inflammatory responses by targeting NADPH oxidase 4 and inhibiting the p38 MAPK pathway [36]. Copper exposure is known to induce oxidative stress and inflammation, which may influence miR-363-3p regulation.

Positive correlations were observed between lead exposure and the expression of hsa-miR-130b-3p, hsa-miR-92a-3p, and hsa-miR-24-3p (*p* < 0.05). Lead is known to induce epigenetic alterations, including changes in miRNA expression, which may contribute to neurotoxicity and immune dysfunction [37]. miR-19a-3p and miR-19b-3p have been reported to be upregulated after spinal cord injury, promoting inflammation by increasing proinflammatory cytokines and reducing the expression of nuclear receptors Nurr1 and Nur77 in microglia [38]. Additionally, miR-130b-3p is implicated in fibroblast regulation by targeting IGF-1 mRNA, which influences epithelial–mesenchymal interactions in lung fibrosis [39]. Given their roles in inflammation and oxidative stress, changes in the expression of these miRNAs may affect cellular responses to lead exposure.

No statistically significant correlation (*p* > 0.05) was found between the Ct values of the seven miRNAs and serum nickel levels. However, miR-24-3p has been shown to be upregulated in dendritic cells (DCs) upon exposure to contact allergens, including nickel, contributing to immune activation [40]. In contrast, our serum-based study found downregulation of miR-24-3p, likely due to differences in tissue-specific expression and exposure context. Nickel exposure is known to cause oxidative stress, inflammation, and fibrosis. Although direct links between nickel and the miRNAs studied are limited, the known functions of miR-19a-3p and miR-130b-3p suggest that their downregulation may worsen the inflammatory and fibrotic effects of nickel exposure.

### 4.5. Occupational and Clinical Implications

Combining occupational exposure assessments with miRNA-based biomarker detection offers a promising strategy for elucidating the genetic factors underlying occupational diseases. This study highlights the potential of circulating miRNAs as effective biomarkers and supports their integration into routine genetic monitoring programs in workplace settings. While the implementation of such programs may involve financial investment, their long-term value in preventing occupational illnesses is substantial. By focusing on seven specific miRNAs, our research aims to identify novel molecular markers that can facilitate the early detection of heavy metal-induced disorders. Moreover, these findings may inform the development of targeted workplace safety regulations and contribute to more effective employee health protection strategies.

### 4.6. Limitations

This study has certain limitations. First, the participants were currently employed and had not been diagnosed with any occupational diseases. They belonged to a workforce with established occupational safety measures and were continuously monitored by an occupational physician. Second, the heavy metal analyses in this study were based on a single blood measurement, conducted using ICP-OES, one of the most reliable methods available. Due to financial constraints, we were unable to validate the results using an alternative measurement technique. Additionally, the analyzed heavy metals have relatively short half-lives in the human body. While blood lead levels in welding workers were higher than those in the control and aluminum-exposed groups, they were not elevated enough to diagnose an occupational disease.

Another limitation was the small sample size. Future studies should include larger cohorts and more comprehensive data collection to enhance the reliability of these findings. Validation in an independent, large-scale cohort is essential to assess the potential of these miRNAs as biomarkers for heavy metal toxicity. Cross-validation with larger datasets will further confirm the consistency and specificity of the observed dysregulation patterns in occupationally exposed workers. Integrating these miRNAs into occupational health surveillance programs could improve the early detection of heavy metal-induced health risks, facilitating timely interventions.

In conclusion, miR-19a-3p, miR-130b-3p, miR-25-3p, miR-363-3p, miR-92a-3p, miR-24-3p, and miR-19b-3p play critical roles in cellular responses to heavy metal exposure. These miRNAs regulate key pathways involved in oxidative stress, inflammation, apoptosis, and DNA damage repair. The dysregulation of these miRNAs upon exposure to toxic metals such as Cr, Cu, Ni, Pb, Al, and Cd underscores their potential as biomarkers for metal-induced toxicity and disease progression. Further research is necessary to elucidate their precise mechanisms and therapeutic applications in mitigating heavy metal-induced health risks.

### 4.7. Broader Applications of miRNA Profiling in Heavy Metal Toxicology

The method used in this study, which combines serum miRNA profiling with bioinformatic gene interaction analyses, could be very useful for other toxicology studies that involve different heavy metals. Beyond aluminum and welding fumes, similar approaches can be extended to study miRNA expression changes in exposure to antimony (Sb), mercury (Hg), and arsenic (As), which are also well-documented environmental toxicants. For example, Sb has been shown to exert toxic effects through oxidative stress and mitochondrial dysfunction, both of which are processes that can alter miRNA expression profiles [41]. Hg, especially in its organic forms, is a strong nerve poison that can disrupt the way genes are controlled, including miRNAs that play a role in brain development and immune system function [42]. Similarly, chronic As exposure has been linked to altered expression of miRNAs associated with carcinogenesis, inflammation, and cardiovascular dysfunction [43]. These findings support the use of circulating miRNAs as accessible and informative biomarkers not only for aluminum and welding fume exposure, but also for a wide range of heavy metal toxicities. Future studies using this methodology could contribute to a more comprehensive understanding of how different metals interact with the genome and epigenome, enabling the development of preventive strategies and early diagnostic tools.

## 5. Conclusions

This pilot study explored the expression patterns of circulating miRNAs in workers occupationally exposed to aluminum and welding fumes, revealing significant downregulation in several miRNAs, including hsa-miR-19a-3p, hsa-miR-130b-3p, hsa-miR-92a-3p, and hsa-miR-24-3p. The integration of serum heavy metal analysis with miRNA expression profiling demonstrated that certain miRNAs—particularly hsa-miR-19a-3p—may serve as potential biomarkers for exposure to aluminum and welding fumes. Weak to moderate correlations between specific metals and miRNA expression were also identified, supporting their involvement in oxidative stress, apoptosis, inflammation, and DNA repair mechanisms.

Moreover, miRNA–target gene network and pathway enrichment analyses highlighted biologically relevant interactions with key regulatory genes such as PTEN, MYC, and TNF. These findings suggest that miRNAs could play a pivotal role in mediating metal-induced cellular effects and may offer valuable insights into the molecular basis of occupational toxicity.

While limited by sample size and the absence of longitudinal data, this study contributes to the growing field of occupational epigenetics by identifying candidate miRNAs for future biomonitoring and risk assessment strategies.

## Figures and Tables

**Table 1:** MIENTURNET enrichment results in Cr, Cu, Ni, Pb, and Al (via miRTarBase).

microRNA	*p*-Value	FDR	Odds Ratio	Number of Interactions	Target Genes
hsa-miR-19a-3p	0.0437	0.33	0.43	6	ESR1, TNF, TF, MYC, ACSL4, PRKN
hsa-miR-130b-3p	0.257	0.523	0.644	4	ESR1, ACSL4, MMP2, IGF1
hsa-miR-25-3p	0.908	0.91	2.34	1	MYC
hsa-miR-363-3p	0.791	0.8	1.54	1	CASP3
hsa-miR-92a-3p	0.959	0.959	2.12	3	CYP7A1, GCHFR, MYC
hsa-miR-24-3p	0.038	0.33	0.482	8	MYC, TGFB1, RRM2, HMOX1, IGF1, IL4, IL1B, TNF
hsa-miR-19b-3p	0.103	0.418	0.537	6	ESR1, LRIG3, ATF2, TGFB1, ACSL4, PRKN

**Table 2:** MIENTURNET enrichment results in Cr, Cu, Ni, Pb, and Al (via TargetScan).

microRNA	*p*-Value	FDR	Odds Ratio	Number of Interactions	Target Genes
hsa-miR-19a-3p/hsa-miR-19b-3p	0.3	0.954	0.778	7	ESR1, LRIG3, ATF2, IGF1, ACSL4, HAVCR1, SLC2A1
hsa-miR-130a-3p	0.408	0.954	0.837	5	IGF1, ESR1, ACSL4, SLC2A1, TNF
hsa-miR-25-3p/hsa-miR-363-3p/hsa-miR-92a-3p	0.988	0.988	4.23	1	NAA15
hsa-miR-24-3p	0.959	0.982	3.09	1	G6PD

**Table 3:** miRNA assay list.

miRNA Symbol	Assay Catolog	Lot Number
hsa-miR-19a-3p	YP00205862	201803080052-4
hsa-miR-19b-3p	YP00204450	31201015-3
hsa-miR-130b-3p	YP00204317	41000618-1
hsa-miR-25-3p	YP00204361	40301621-2
hsa-miR-363-3p	YP00204726	201803060217-1
hsa-miR-92a-3p	YP00204258	40805431-3
hsa-miR-24-3p	YP00204260	31200624-2
hsa-miR-16-5p	YP00205702	40501706-1

**Table 4:** PCR cycling conditions.

Cycles	Duration	Temperature	Program Name
1	2 min	95 °C	PCR initial heat activation
45	15 s	95 °C	Denaturation
	60 s	56 °C	Combined annealing/extension
1		60 °C 95 °C	Melting curve analysis

**Table 5:** Age, working years, and smoking distribution of the people included in the study.

Descriptives		n	Mean	Std. Deviation	95% CI		Minimum	Maximum	Significance	
					Lower	Upper			F	*p*
Age (Year)	Control Group	16	39.69	6.39	36.29	43.09	32	55	1.079	0.348
	Aluminum workers	16	38.69	10.35	33.17	44.20	20	55		
	Welding workers	16	35.06	10.75	29.34	40.79	20	54		
Year of work	Control Group	16	10.88	2.68	9.45	12.30	7	16	0.241	0.787
	Aluminum workers	16	10.50	3.25	8.77	12.23	2	15		
	Welding workers	16	11.50	5.75	8.44	14.56	1	18		
Smoking/pack/year	Control Group	16	4.81	1.76	3.87	5.75	2	8	2.227	0.12
	Aluminum workers	16	5.38	2.03	4.29	6.46	2	10		
	Welding workers	16	6.31	2.27	5.10	7.52	2	10		

**Table 6:** Metal concentration (mg/L) in controls, aluminum workers, and welding workers.

		Control					Aliminum Workers					Welding Workers			
Descriptive	Al	Cr	Cu	Ni	Pb	Al *	Cr	Cu	Ni	Pb ^¶^	Al *	Cr	Cu	Ni	Pb ^¶^
Mean	4.932	nd	3.001	2.976	0.667	7.320	0.237	3.099	3.347	0.804	6.904	0.316	3.134	3.196	0.903
Std. Deviation	0.566	nd	0.165	0.342	0.068	1.630	0.191	0.334	1.264	0.104	1.998	0.145	0.168	1.500	0.084
Median	4.881	nd	3.025	2.900	0.653	6.980	0.172	3.183	2.896	0.824	6.489	0.298	3.130	2.632	0.890
Minimum	4.240	nd	2.722	2.479	0.580	5.620	0.046	2.456	2.279	0.574	4.357	0.075	2.903	2.332	0.719
Maximum	5.810	nd	3.307	3.553	0.821	12.429	0.654	3.610	7.567	0.930	12.952	0.554	3.442	8.343	1.053

Cadmium was not detected in all three groups; * *p* < 0.001 is significantly higher compared to that in the control group; ^¶^ significantly higher than the control group; nd—not detected.

**Table 7:** Correlation coefficients for miRNAs analyzed in relation to heavy metal results.

	Heavy Metals	hsa-miR-19a-3p	hsa-miR-19b-3p	hsa-miR-130b-3p	hsa-miR-25-3p	hsa-miR-363-3p	hsa-miR-92a-3p	hsa-miR-24-3p
Al	Pearson Correlation	0.319 *	0.413 **	0.356 *	0.304 *	0.183	0.354 *	0.411 **
	*p*	0.027	0.003	0.013	0.036	0.214	0.014	0.004
	n	48	48	48	48	48	48	48
Cr	Pearson Correlation	−0.225	−0.156	0.255	−0.126	0.383 *	0.363 *	0.245
	*p*	0.216	0.393	0.16	0.49	0.03	0.041	0.177
	n	32	32	32	32	32	32	32
Cu	Pearson Correlation	0.075	0.093	−0.046	0.195	−0.295 *	−0.089	−0.057
	*p*	0.611	0.531	0.756	0.185	0.042	0.549	0.701
	n	48	48	48	48	48	48	48
Ni	Pearson Correlation	−0.018	0.091	0.074	−0.006	0.047	0.032	0.014
	*p*	0.902	0.537	0.616	0.967	0.753	0.829	0.923
	n	48	48	48	48	48	48	48
Pb	Pearson Correlation	0.175	0.112	0.399 **	0.006	0.209	0.370 **	0.478 **
	*p*	0.233	0.447	0.005	0.965	0.154	0.01	0.001
	n	48	48	48	48	48	48	48

* *p* < 0.05; ** *p* < 0.01.

**Table 8:** Comparison of mean ΔCt and fold change values between the heavy metal-exposed group and the group without heavy metal exposure (*p* < 0.05).

miRNA	ΔCt			ΔΔCt			2^−ΔΔCt^			Fold Change		Fold Up or Down-Regulation		*p* Value	*p* Value
	AE	WFE	Non-E	AE	WFE	Non-E	AE	WFE	Non-E	AE/ Non-E	WFE/ Non-E	AE/ Non-E	WFE/ Non-E	AE/ Non-E	WFE/ Non-E
hsa-miR-19a-3p	20.51	18.34	21.02	1.84375	1.61875	1.18125	0.278597	0.325617	0.440969	0.6318	0.7384	−1.5828	−1.3543	0.009145	0.01051
hsa-miR-19b-3p	21.12	18.64	21.72	2.456875	1.915	1.699375	0.182141	0.265172	0.307919	0.5915	0.8612	−1.6906	−1.1612	0.010407	0.243103
hsa-miR-130b-3p	26.09	24.77	26.83	7.42625	8.043125	6.80375	0.005814	0.003791	0.008951	0.6495	0.4236	−1.5395	−2.361	0.572081	0.000041
hsa-miR-25-3p	22.46	20.2	23.4	3.79375	3.47375	3.376875	0.072105	0.090011	0.096263	0.749	0.9351	−1.335	−1.0695	0.283009	0.547976
hsa-miR-363-3p	24.89	23.24	26.23	6.22625	6.515625	6.205	0.013357	0.01093	0.013555	0.9854	0.8063	−1.0148	−1.2402	0.636084	0.08771
hsa-miR-92a-3p	20.16	18.81	21.29	1.491875	2.09	1.269375	0.35555	0.234881	0.414839	0.8571	0.5662	−1.1668	−1.7662	0.849347	0.000375
hsa-miR-24-3p	22.35	21.13	23.13	3.68625	4.4125	3.11	0.077683	0.046958	0.115824	0.6707	0.4054	−1.491	−2.4666	0.098174	*p* < 0.001

ΔCt—delta cycle threshold; AE—aluminum-exposed; WFE—welding fume-exposed; Non-E—non-exposed; ΔΔCt—Delta-Delta Ct.

**Table 9:** ΔCt values in the control group according to working years.

Groups	miRNA	Years of Working	n	Mean	Std. Deviation	Minimum	Maximum	z	Sig.
Control Group	ΔCt hsa-miR-19a-3p	10 years and under	10	1.30	0.36	0.90	1.94	−0.651	0.515
		11 years and above	6	0.98	0.70	0.12	1.71		
	ΔCt hsa-miR-19b-3p	10 years and under	10	1.74	0.54	0.60	2.38	−0.434	0.664
		11 years and above	6	1.63	0.45	1.03	2.01		
	ΔCt hsa-miR-130b-3p	10 years and under	10	6.62	0.78	5.85	8.12	−1.41	0.159
		11 years and above	6	7.11	0.76	6.09	8.17		
	ΔCt hsa-miR-25-3p	10 years and under	10	3.42	0.45	2.69	4.36	−0.76	0.447
		11 years and above	6	3.31	0.24	3.06	3.68		
	ΔCt hsa-miR-363-3p	10 years and under	10	6.16	0.97	4.53	8.28	−0.651	0.515
		11 years and above	6	6.29	0.24	6.04	6.67		
	ΔCt hsa-miR-92a-3p	10 years and under	10	1.19	0.58	−0.10	2.12	−0.651	0.515
		11 years and above	6	1.40	0.62	0.60	2.17		
	ΔCt hsa-miR-24-3p	10 years and under	10	2.91	0.34	2.49	3.44	−2.118	0.034
		11 years and above	6	3.45	0.49	2.87	4.24		

**Table 10:** ΔCt values in the aluminum group according to the working years.

Groups	miRNA	Years of Working	n	Mean	Std. Deviation	Minimum	Maximum	z	Sig.
Aluminum workers	ΔCt hsa-miR-19a-3p	10 years and under	9	1.89	0.92	0.96	3.58	0	1
		11years and above	7	1.78	0.45	1.05	2.57		
	ΔCt hsa-miR-19b-3p	10 years and under	9	2.50	0.93	0.71	3.97	−0.159	0.874
		11 years and above	7	2.40	0.43	2.04	3.18		
	ΔCt hsa-miR-130b-3p	10 years and under	9	7.34	1.50	5.62	9.94	−0.318	0.751
		11 years and above	7	7.53	1.66	5.25	10.07		
	ΔCt hsa-miR-25-3p	10 years and under	9	3.94	1.43	2.35	6.49	−0.742	0.458
		11 years and above	7	3.61	0.26	3.33	4.05		
	ΔCt hsa-miR-363-3p	10 years and under	9	6.00	1.12	3.36	7.41	−0.212	0.832
		11 years and above	7	6.52	0.86	5.67	7.90		
	ΔCt hsa-miR-92a-3p	10 years and under	9	1.36	0.88	-0.30	2.91	−0.371	0.711
		11 years and above	7	1.67	1.11	0.12	3.59		
	ΔCt hsa-miR-24-3p	10 years and under	9	3.34	0.73	2.24	4.10	−1.115	0.265
		11 years and above	7	4.13	1.28	2.77	6.80		

**Table 11:** ΔCt values according to working years in the welding workers group.

Groups	miRNA	Years of Working	n	Mean	Std. Deviation	Minimum	Maximum	z	Sig.
Welding workers	ΔCt hsa-miR-19a-3p	10 years and under	6	1.65	0.38	1.10	2.15	−0.435	0.664
		11 years and above	10	1.60	0.23	1.10	1.83		
	ΔCt hsa-miR-19b-3p	10 years and under	6	1.76	0.60	0.72	2.29	−0.542	0.588
		11 years and above	10	2.01	0.30	1.52	2.44		
	ΔCt hsa-miR-130b-3p	10 years and under	6	8.20	0.87	6.91	8.96	−0.759	0.448
		11 years and above	10	7.95	0.33	7.53	8.53		
	ΔCt hsa-miR-25-3p	10 years and under	6	3.29	0.50	2.39	3.70	−1.303	0.193
		11 years and above	10	3.58	0.24	3.12	3.87		
	ΔCt hsa-miR-363-3p	10 years and under	6	6.25	0.30	5.76	6.66	−2.386	0.017
		11 years and above	10	6.68	0.33	5.88	7.04		
	ΔCt hsa-miR-92a-3p	10 years and under	6	2.02	0.35	1.34	2.26	−0.326	0.745
		11 years and above	10	2.13	0.18	1.88	2.51		
	ΔCt hsa-miR-24-3p	10 years and under	6	4.72	0.84	3.53	5.77	−1.193	0.233
		11 years and above	10	4.23	0.42	3.65	4.84		

**Table 12:** ΔCt values in the aluminum workers group based on smoking years.

Groups	miRNA	Years of Smoking	n	Mean	Std. Deviation	Minimum	Maximum	z	Sig.
Control Group	ΔCt hsa-miR-19a-3p	Under 5 packs years	10	1.14	0.60	0.12	1.94	0	1
		Over 5 packs years	6	1.25	0.39	0.90	1.74		
	ΔCt hsa-miR-19b-3p	Under 5 packs years	10	1.71	0.43	1.03	2.38	−0.217	0.828
		Over 5 packs years	6	1.69	0.64	0.60	2.37		
	ΔCt hsa-miR-130b-3p	Under 5 packs years	10	6.79	0.81	5.85	8.12	−0.325	0.745
		Over 5 packs years	6	6.83	0.80	5.98	8.17		
	ΔCt hsa-miR-25-3p	Under 5 packs years	10	3.41	0.40	2.97	4.36	−0.163	0.871
		Over 5 packs years	6	3.33	0.38	2.69	3.70		
	ΔCt hsa-miR-363-3p	Under 5 packs years	10	6.09	0.61	4.53	6.67	−0.054	0.957
		Over 5 packs years	6	6.41	1.00	5.52	8.28		
	ΔCt hsa-miR-92a-3p	Under 5 packs years	10	1.47	0.52	0.85	2.17	−1.193	0.233
		Over 5 packs years	6	0.94	0.58	−0.10	1.36		
	ΔCt hsa-miR-24-3p	Under 5 packs years	10	3.12	0.58	2.49	4.24	−0.217	0.828
		Over 5 packs years	6	3.10	0.25	2.60	3.24		

**Table 13:** ΔCt values in the aluminum workers group according to smoking years.

Groups	miRNA	Years of Smoking	n	Mean	Std. Deviation	Minimum	Maximum	z	Sig.
Aluminum workers	ΔCt hsa-miR-19a-3p	Under 5 packs years	9	1.69	0.80	0.96	3.58	−1.218	0.223
		Over 5 packs years	7	2.04	0.64	1.05	3.02		
	ΔCt hsa-miR-19b-3p	Under 5 packs years	9	2.24	0.72	0.71	3.28	−1.166	0.244
		Over 5 packs years	7	2.74	0.68	2.04	3.97		
	ΔCt hsa-miR-130b-3p	Under 5 packs years	9	6.98	1.18	5.62	9.29	−1.218	0.223
		Over 5 packs years	7	8.00	1.80	5.25	10.07		
	ΔCt hsa-miR-25-3p	Under 5 packs years	9	3.59	1.06	2.35	6.24	−1.536	0.125
		Over 5 packs years	7	4.06	1.10	3.33	6.49		
	ΔCt hsa-miR-363-3p	Under 5 packs years	9	5.97	1.05	3.36	6.85	−0.053	0.958
		Over 5 packs years	7	6.55	0.95	5.67	7.90		
	ΔCt hsa-miR-92a-3p	Under 5 packs years	9	1.29	0.75	−0.30	2.20	−0.371	0.711
		Over 5 packs years	7	1.75	1.20	0.12	3.59		
	ΔCt hsa-miR-24-3p	Under 5 packs years	9	3.34	0.73	2.24	4.10	−1.327	0.184
		Over 5 packs years	7	4.14	1.27	2.77	6.80		

**Table 14:** ΔCt values in the welding workers group according to smoking years.

Groups	miRNA	Years of Smoking	n	Mean	Std. Deviation	Minimum	Maximum	z	Sig.
Welding workers	ΔCt hsa-miR-19a-3p	Under 5 packs years	6	1.64	0.34	1.10	2.15	−0.054	0.957
		Over 5 packs years	10	1.61	0.26	1.10	1.88		
	ΔCt hsa-miR-19b-3p	Under 5 packs years	6	1.61	0.49	0.72	2.11	−2.278	0.023
		Over 5 packs years	10	2.10	0.29	1.49	2.44		
	ΔCt hsa-miR-130b-3p	Under 5 packs years	6	7.73	0.44	6.91	8.25	−1.41	0.159
		Over 5 packs years	10	8.23	0.58	7.39	8.96		
	ΔCt hsa-miR-25-3p	Under 5 packs years	6	3.20	0.47	2.39	3.72	−2.171	0.03
		Over 5 packs years	10	3.64	0.17	3.42	3.87		
	ΔCt hsa-miR-363-3p	Under 5 packs years	6	6.40	0.50	5.76	7.04	−0.651	0.515
		Over 5 packs years	10	6.59	0.28	6.12	6.98		
	ΔCt hsa-miR-92a-3p	Under 5 packs years	6	1.94	0.34	1.34	2.30	−1.194	0.232
		Over 5 packs years	10	2.18	0.15	1.91	2.51		
	ΔCt hsa-miR-24-3p	Under 5 packs years	6	4.18	0.21	3.88	4.50	−1.085	0.278
		Over 5 packs years	10	4.55	0.76	3.53	5.77		

**Table 15:** Group and study year interaction in ΔCt hsa-miR-19a-3p.

Tests of Between-Subjects Effects					
Dependent Variable: ΔCt hsa-miR-19a-3p					
Source	Type III Sum of Squares	df	Mean Square	F	Sig.
Corrected Model	4.078a	5	0.816	2.684	0.034
Intercept	107.591	1	107.591	354.084	*p* < 0.001
Groups	3.888	2	1.944	6.398	0.004
YearOfWork	0.283	1	0.283	0.933	0.34
Groups * YearOfWork	0.165	2	0.083	0.272	0.763
Error	12.762	42	0.304		
Total	131.85	48			
Corrected Total	16.84	47			
a R Squared = 0.242 (Adjusted R Squared = 0.152)					

**Table 16:** Group and smoking year interaction in ΔCt hsa-miR-19a-3p.

Dependent Variable: ΔCt hsa-miR-19a-3p					
Source	Type III Sum of Squares	df	Mean Square	F	Sig.
Corrected Model	4.164a	5	0.833	2.759	0.03
Intercept	111.479	1	111.479	369.37	*p* < 0.001
Groups	3.524	2	1.762	5.837	0.006
Smoking	0.229	1	0.229	0.76	0.388
Groups * Smoking	0.291	2	0.146	0.483	0.62
Error	12.676	42	0.302		
Total	131.85	48			
Corrected Total	16.84	47			
a R Squared = 0.247 (Adjusted R Squared = 0.158)					

**Table 17:** Group and study year interaction in ΔCt hsa-miR-19b-3p.

Tests of Between-Subjects Effects					
Dependent Variable: ΔCt hsa-miR-19b-3p					
Source	Type III Sum of Squares	df	Mean Square	F	Sig.
Corrected Model	5.188a	5	1.038	3.056	0.019
Intercept	184.116	1	184.116	542.354	*p* < 0.001
Groups	4.889	2	2.444	7.201	0.002
YearOfWork	0.001	1	0.001	0.004	0.95
Groups * YearOfWork	0.312	2	0.156	0.46	0.634
Error	14.258	42	0.339		
Total	216.033	48			
Corrected Total	19.446	47			
a R Squared = 0.267 (Adjusted R Squared = 0.179)					

**Table 18:** Group and smoking year interaction in ΔCt hsa-miR-19b-3p.

Dependent Variable: ΔCt hsa-miR-19b-3p					
Source	Type III Sum of Squares	df	Mean Square	F	Sig.
Corrected Model	6.795a	5	1.359	4.512	0.002
Intercept	185.309	1	185.309	615.25	*p* < 0.001
Groups	5.434	2	2.717	9.02	0.001
Smoking	1.23	1	1.23	4.085	0.05
Groups * Smoking	0.668	2	0.334	1.109	0.339
Error	12.65	42	0.301		
Total	216.033	48			
Corrected Total	19.446	47			
a R Squared = 0.349 (Adjusted R Squared = 0.272)					

**Table 19:** Group and study year interaction in ΔCt hsa-miR-24-3p.

Dependent Variable: ΔCt hsa-miR-24-3p					
Source	Type III Sum of Squares	df	Mean Square	F	Sig.
Corrected Model	18.047a	5	3.609	7.102	*p* < 0.001
Intercept	658.726	1	658.726	1296.073	*p* < 0.001
Groups	12.695	2	6.347	12.489	*p* < 0.001
YearOfWork	0.876	1	0.876	1.724	0.196
Groups * YearOfWork	3.485	2	1.742	3.428	0.042
Error	21.346	42	0.508		
Total	709.452	48			
Corrected Total	39.393	47			
a R Squared = 0.458 (Adjusted R Squared = 0.394)					

**Table 20:** Group and smoking year interaction in ΔCt hsa-miR-24-3p.

Dependent Variable: ΔCt hsa-miR-24-3p					
Source	Type III Sum of Squares	df	Mean Square	F	Sig.
Corrected Model	16.675a	5	3.335	6.165	*p* < 0.001
Intercept	638.494	1	638.494	1180.397	*p* < 0.001
Groups	11.882	2	5.941	10.983	*p* < 0.001
Smoking	1.686	1	1.686	3.118	0.085
Groups * Smoking	1.295	2	0.647	1.197	0.312
Error	22.718	42	0.541		
Total	709.452	48			
Corrected Total	39.393	47			
a R Squared = 0.423 (Adjusted R Squared = 0.355)					

**Table 21:** Group and study year interaction in ΔCt hsa-miR-92a-3p.

Dependent Variable: hsa-miR-92a-3p					
Source	Type III Sum of Squares	df	Mean Square	F	Sig.
Corrected Model	6.353a	5	1.271	2.728	0.032
Intercept	121.109	1	121.109	260.073	*p* < 0.001
Groups	4.879	2	2.44	5.239	0.009
YearOfWork	0.508	1	0.508	1.092	0.302
Groups * YearOfWork	0.073	2	0.036	0.078	0.925
Error	19.558	42	0.466		
Total	151.429	48			
Corrected Total	25.911	47			
a R Squared = 0.245 (Adjusted R Squared = 0.155)					

**Table 22:** Group and smoking year interaction in ΔCt hsa-miR-92a-3p.

Dependent Variable: ΔCt hsa-miR-92a-3p					
Source	Type III Sum of Squares	df	Mean Square	F	Sig.
Corrected Model	7.880a	5	1.576	3.671	0.008
Intercept	116.297	1	116.297	270.897	*p* < 0.001
Groups	5.646	2	2.823	6.576	0.003
Smoking	0.035	1	0.035	0.082	0.776
Groups * Smoking	2.073	2	1.036	2.414	0.102
Error	18.031	42	0.429		
Total	151.429	48			
Corrected Total	25.911	47			
a R Squared = 0.304 (Adjusted R Squared = 0.221)					

**Table 23:** Group and study year interaction in ΔCt hsa-miR-363-3p.

Dependent Variable: ΔCt hsa-miR-363-3p					
Source	Type III Sum of Squares	df	Mean Square	F	Sig.
Corrected Model	2.783a	5	0.557	0.948	0.461
Intercept	1822.848	1	1822.848	3103.541	*p* < 0.001
Groups	0.506	2	0.253	0.43	0.653
YearOfWork	1.476	1	1.476	2.513	0.12
Groups * YearOfWork	0.321	2	0.16	0.273	0.762
Error	24.668	42	0.587		
Total	1942.033	48			
Corrected Total	27.451	47			
a R Squared = 0.101 (Adjusted R Squared = −0.006)					

**Table 24:** Group and smoking year interaction in ΔCt hsa-miR-363-3p.

Dependent Variable: ΔCt hsa-miR-363-3p					
Source	Type III Sum of Squares	df	Mean Square	F	Sig.
Corrected Model	2.811a	5	0.562	0.958	0.454
Intercept	1833.992	1	1833.992	3126.066	*p* < 0.001
Groups	0.573	2	0.286	0.488	0.617
Smoking	1.512	1	1.512	2.577	0.116
Groups * Smoking	0.305	2	0.153	0.26	0.772
Error	24.64	42	0.587		
Total	1942.033	48			
Corrected Total	27.451	47			
a R Squared = 0.102 (Adjusted R Squared = −0.004)					

**Table 25:** Group and study year interaction in ΔCt hsa-miR-130b-3p.

Dependent Variable: ΔCt hsa-miR-130b-3p.					
Source	Type III Sum of Squares	df	Mean Square	F	Sig.
Corrected Model	13.546a	5	2.709	2.386	0.054
Intercept	2544.052	1	2544.052	2240.406	*p* < 0.001
Groups	11.006	2	5.503	4.846	0.013
YearOfWork	0.224	1	0.224	0.198	0.659
Groups * YearOfWork	1.03	2	0.515	0.454	0.638
Error	47.692	42	1.136		
Total	2707.063	48			
Corrected Total	61.239	47			
a R Squared = 0.221 (Adjusted R Squared = 0.128)					

**Table 26:** Group and smoking year interaction in ΔCt hsa-miR-130b-3p.

Dependent Variable: ΔCt hsa-miR-130b-3p					
Source	Type III Sum of Squares	df	Mean Square	F	Sig.
Corrected Model	17.363a	5	3.473	3.324	0.013
Intercept	2521.983	1	2521.983	2414.175	*p* < 0.001
Groups	10.399	2	5.2	4.977	0.011
Smoking	3.102	1	3.102	2.969	0.092
Groups * Smoking	1.872	2	0.936	0.896	0.416
Error	43.876	42	1.045		
Total	2707.063	48			
Corrected Total	61.239	47			
a R Squared = 0.284 (Adjusted R Squared = 0.198)					

**Table 27:** Area under the curve values and their significance for aluminum.

MiRNAs	AUC	Std. Error	Significance	95%CI	
				Lower Bound	Upper Bound
hsa-miR-19a-3p	0.764	0.084	0.011	0.599	0.929
hsa-miR-19b-3p	0.852	0.072	0.001	0.710	0.993

**Table 28:** Sensitivity and specificity values of aluminum workers compared to the control group.

MiRNAs	Positive if Greater Than or Equal to	Sensitivity	1—Specificity	Specificity	Sensitivity + Specificity
hsa-miR-19a-3p	1.47	0.69	0.31	0.69	1.38
	1.59	0.69	0.25	0.75	1.44
hsa-miR-19b-3p	2.03	0.88	0.19	0.81	1.69
	2.08	0.75	0.19	0.81	1.56

**Table 29:** Area under the curve values and their significance.

MiRNAs	Area AUC	Std. Error	Significance	95%CI	
				Lower Bound	Upper Bound
hsa-miR-19a-3p	0.773	0.084	0.008	0.608	0.939
hsa miR-130b-3p	0.898	0.055	*p* < 0.001	0.790	1.000
hsa miR-92a-3p	0.914	0.049	*p* < 0.001	0.817	1.000
hsa miR-24-3p	0.961	0.032	*p* < 0.001	0.898	1.000

**Table 30:** Sensitivity and specificity values of welding workers compared to the control group.

MiRNA	Positive if Greater Than or Equal to	Sensitivity	1—Specificity	Specificity	Sensitivity + Specificity
hsa miR-19a-3p	1.39	0.81	0.31	0.69	1.50
	1.44	0.75	0.31	0.69	1.44
hsa-miR-130b-3p	7.61	0.81	0.19	0.81	1.63
	7.67	0.81	0.13	0.88	1.69
hsa-miR-92a-3p	1.57	0.94	0.25	0.75	1.69
	1.83	0.94	0.19	0.81	1.75
hsa-miR-24-3p	6.39	0.69	0.25	0.75	1.44
	6.46	0.63	0.25	0.75	1.38

## Data Availability

Data are contained within the article and Supplementary Materials.

## Supplementary Materials

The following supporting information can be downloaded at: https://www.mdpi.com/article/10.3390/cimb47050306/s1.
