# Supplementary material for: A Pilot Investigation of Circulating miRNA Expression in Individuals Exposed to Aluminum and Welding Fumes
Source: Curr Issues Mol Biol. 2025 Apr 26;47(5):306. doi: 10.3390/cimb47050306 (PMC12109762; doi:10.3390/cimb47050306)
Supplement: Supplementary file 1 [file cimb-47-00306-s001.zip › cimb-3584528-supplementary.pdf]

# Supplementary Analysis: Reference Gene Comparison in qPCR Normalization

This document provides extended analyses supporting the results and reference gene justification presented in the main manuscript. Both hsa-miR-16-5p and RNU6 (U6 snRNA) were used for normalization in qRT-PCR analyses.

## 1. Reference Gene Stability Analysis

The cycle threshold (Ct) values of both reference genes were evaluated across all 48 samples (16 controls, 16 AE, 16 WFE). miR-16-5p displayed a mean Ct of 18.5 with a standard deviation (SD) of 1.78, while U6 had a higher mean Ct of 32.7 and SD of 1.80. Lower Ct values are generally more reliable for qPCR normalization, indicating miR-16-5p may be more stable and efficient under the experimental conditions.

## 2. Comparison of Differentially Expressed miRNAs

The selection of reference gene significantly influenced the list of differentially expressed miRNAs. Venn diagrams below illustrate the overlap between significant miRNAs when normalized to miR-16-5p and U6 in both AE and WFE groups.

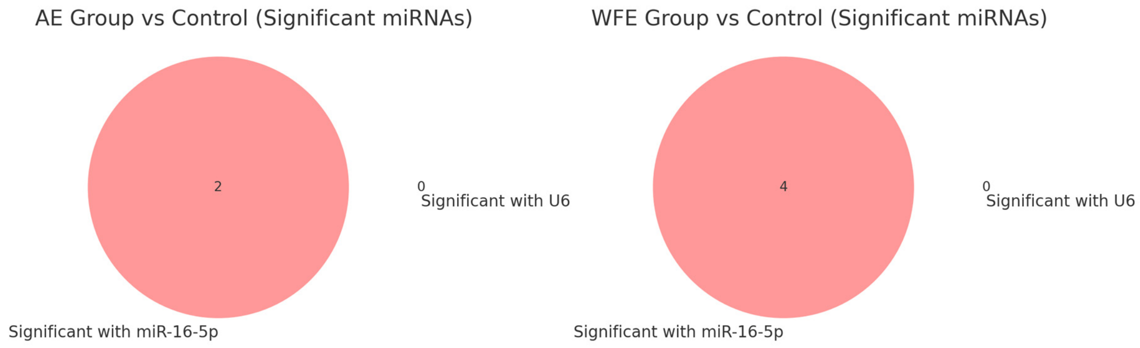


**Supplementary Figure S1.** Venn diagrams comparing significant miRNAs detected using miR-16-5p and U6. The overlap is minimal, indicating reference gene choice alters biological conclusions.

## 3. Heatmap of Fold Changes (log2)

A heatmap of log2-transformed fold changes shows the relative expression levels of miRNAs across groups and reference genes. Only miRNAs with complete data are shown.

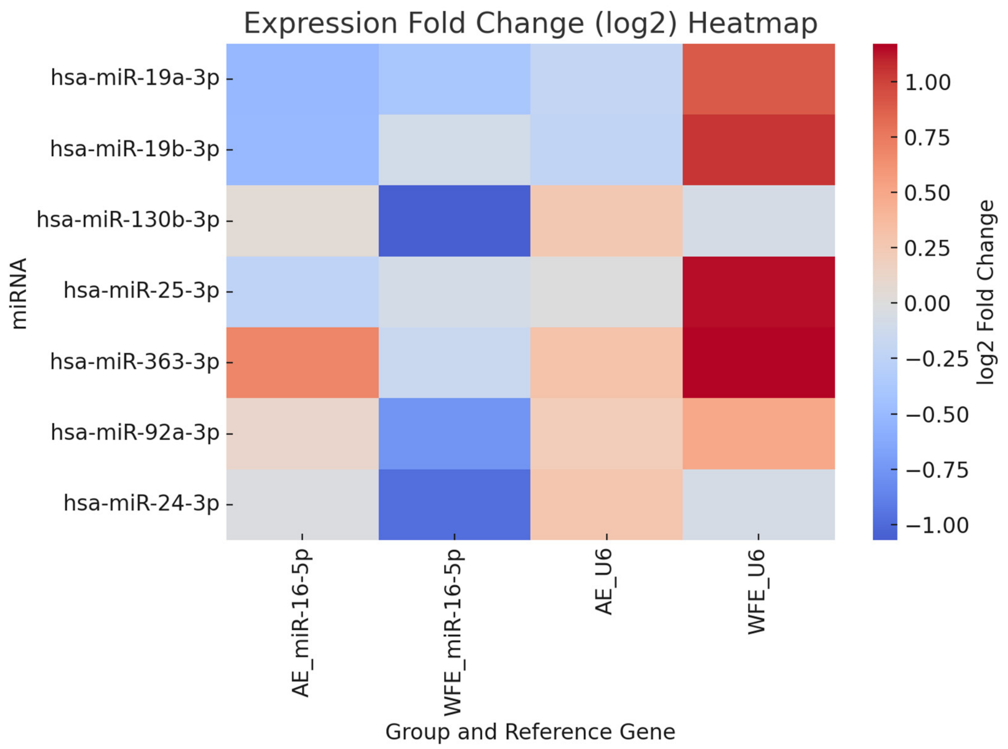


**Supplementary Figure S2**. Heatmap of log2 fold changes in AE and WFE groups normalized to miR-16-5p and U6. Red indicates upregulation, blue indicates downregulation.

## 4. Fold Change Scatterplot

Fold changes computed using both reference genes were plotted for each miRNA. A diagonal reference line indicates agreement. Deviations from this line show inconsistencies caused by reference gene selection.

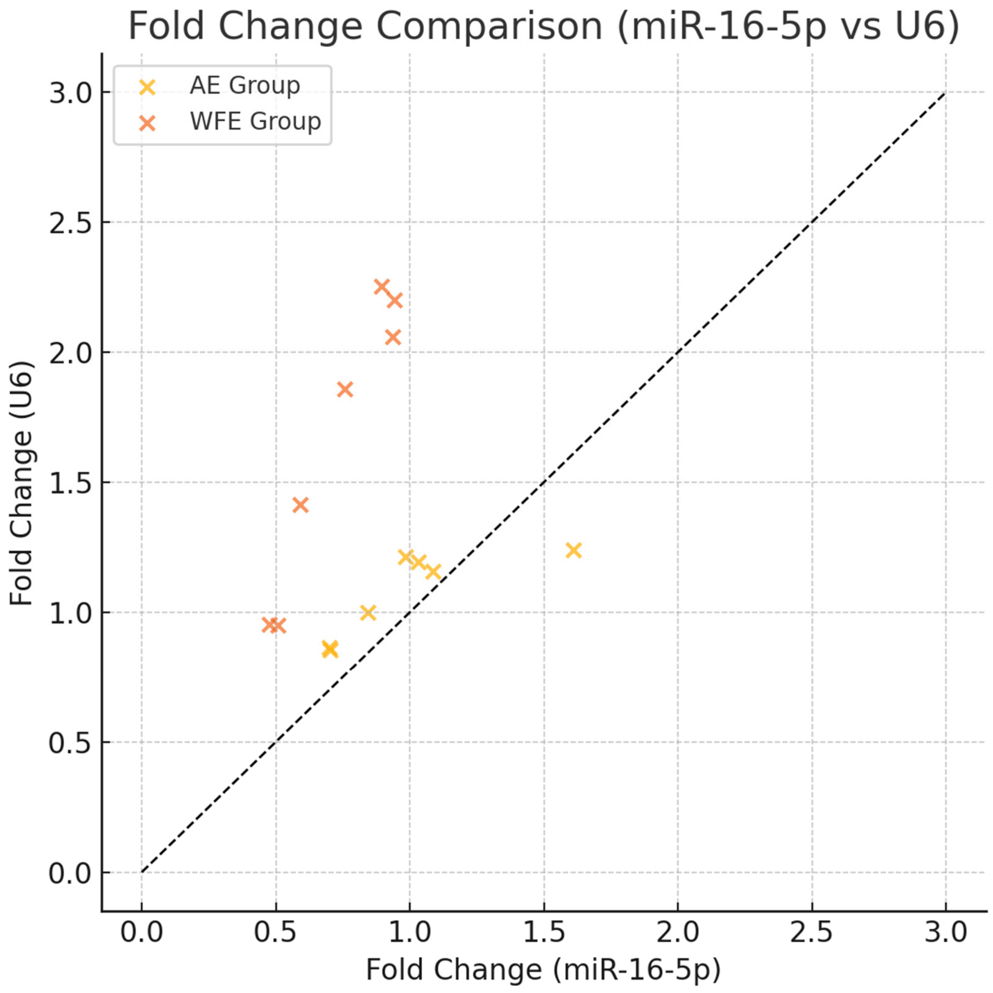


**Supplementary Figure S3.** Comparison of fold changes using miR-16-5p vs. U6 in AE and WFE groups. Points off the diagonal indicate reference gene-dependent differences.

## 5. Volcano Plot of AE Group (miR-16-5p)

Volcano plots visualize statistical significance and biological relevance. Points marked with an 'X' represent miRNAs with log2 fold change > 1 and p-value < 0.05 (highlighted threshold lines). These are considered biologically and statistically significant.

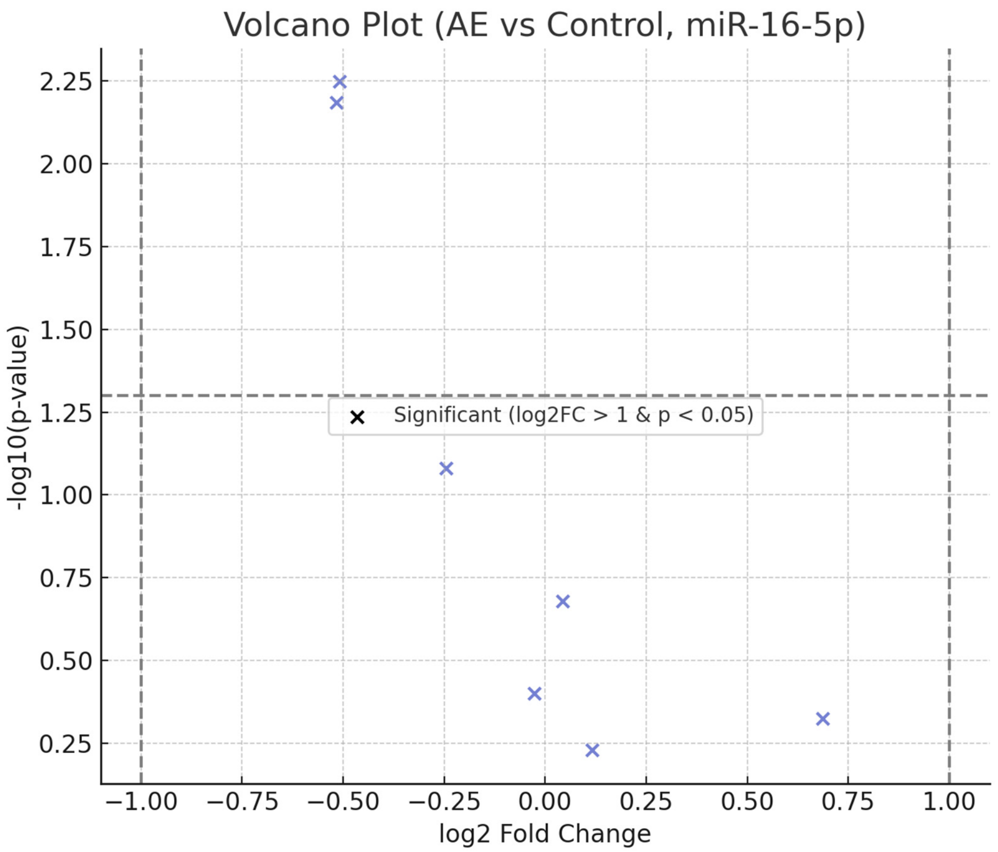


**Supplementary Figure S4.** Volcano plot for AE vs. Control using miR-16-5p. X-marked miRNAs show both statistical significance ($p < 0.05$) and biological relevance (log2FC > 1).

## Conclusion

This extended analysis underscores the importance of validating reference genes in each experimental context. Our results indicate that miR-16-5p is more suitable for normalization due to its lower Ct, greater stability, and consistency with the expression range of target miRNAs. The choice of reference gene significantly affects which miRNAs appear significantly dysregulated, highlighting the necessity for careful selection and transparent reporting of normalization strategies.

## 6. Limitations of U6 snRNA as a Reference Gene in Serum

Although U6 snRNA is widely used as a reference gene in cellular miRNA studies, growing evidence suggests that it may not be suitable for normalization in serum-based miRNA profiling. Zhao J et al. (2020) (https://doi.org/10.7150/jca.48903) reported that U6 is rapidly degraded in serum, making it unreliable for quantifying circulating miRNAs. Benz F et al. (2013) (https://doi.org/10.1038/emm.2013.81) demonstrated that U6 snRNA levels in serum are highly variable and subject to disease-specific regulation, showing significant upregulation in patients with sepsis and critical illness and marked downregulation in liver fibrosis. Their findings provide clear evidence that U6 is not a stable or suitable reference gene for normalization in serum-based miRNA studies, due to its interindividual variability and sensitivity to pathological conditions. Taken together, these findings strongly suggest that U6 is not a robust normalizer in extracellular contexts such as serum or plasma. Alternative miRNAs, including miR-16-5p, miR-191-5p, or miR-103a-3p, are generally more stable in such environments and may provide more reliable results for RT-qPCR normalization.
